# Internal Versus External Motivation in Referral of Primary Care Patients with Depression to an Internet Support Group: Randomized Controlled Trial

**DOI:** 10.2196/jmir.2197

**Published:** 2013-03-12

**Authors:** Benjamin W Van Voorhees, Robert C Hsiung, Monika Marko-Holguin, Thomas K Houston, Joshua Fogel, Royce Lee, Daniel E Ford

**Affiliations:** ^1^Section of General Pediatrics Department of PediatricsUniversity of Illinois at ChicagoChicago, ILUnited States; ^2^Dr. Bob LLCChicago, ILUnited States; ^3^Division of Health Informatics and Implementation ScienceUniversity of Massachusetts Medical SchoolWorcester, MAUnited States; ^4^eHealth QUERIBedford VA Medical CenterBedford, MAUnited States; ^5^Department of Finance and Business ManagementBrooklyn College of the City University of New YorkBrooklyn, NYUnited States; ^6^Department of PsychiatryThe University of ChicagoChicago, ILUnited States; ^7^Division of General Internal MedicineDepartment of MedicineJohns Hopkins Medical InstitutionsBaltimore, MDUnited States

**Keywords:** depressive disorder, Internet, primary care, support groups

## Abstract

**Background:**

Depressive disorders and symptoms affect more than one-third of primary care patients, many of whom do not receive or do not complete treatment. Internet-based social support from peers could sustain depression treatment engagement and adherence. We do not know whether primary care patients will accept referral to such websites nor do we know which methods of referral would be most effective.

**Objective:**

We conducted a randomized clinical trial to determine whether (1) a simple generic referral card (control), (2) a patient-oriented brochure that provided examples of online postings and experience (internal motivation), or (3) a physician letter of recommendation (external motivation) would generate the greatest participation in a primary care Internet depression treatment support portal focused around an Internet support group (ISG).

**Methods:**

We used 3 offline methods to identify potential participants who had not used an ISG in the past 6 months. Eligibility was determined in part by a brief structured psychiatric interview based on the Patient Health Questionnaire-9 (PHQ-9). After consent and enrollment, participants were randomly assigned to 1 of 3 groups (control, internal motivation, or external motivation). We constructed a portal to connect primary care patients to both fact-based information and an established ISG (Psycho-Babble). The ISG allowed participants to view messages and then decide if they actually wished to register there. Participation in the portal and the ISG was assessed via automated activity tracking.

**Results:**

Fifty participants were assigned to the 3 groups: a motivation-neutral control group (n=18), an internal motivation group (n=19), and an external motivation group (n=13). Of these participants, 31 (62%) visited the portal; 27 (54%) visited the ISG itself. The internal motivation group showed significantly greater participation than the control group on several measures. The external motivation group spent significantly less time logged onto the portal than the control group. The internal motivation group showed significantly greater participation than the external motivation group on several measures.

**Conclusions:**

Referral of primary care patients with depressive disorders and symptoms to an ISG is feasible even if they have never previously used one. This may best be accomplished by enhancing their internal motivation.

**Trial Registration:**

Clinicaltrials.gov: NCT00886730; http://clinicaltrials.gov/show/NCT00886730 (Archived by WebCite at http://www.webcitation.org/6F4981fDN)

## Introduction

Mood disorders have a lifetime prevalence of 20.8% [1-3]. Many Americans with a depressive disorder do not receive any treatment, and many of those who do receive treatment either do not receive high quality treatment or do not complete a full course of treatment [4]. Chronic care and collaborative care models have demonstrated benefit in improving process of care, symptoms, and functional outcomes [5-7]. A key component of these models appears to be support provided by case management, which may act by improving adherence or increasing patient activation [6,8]. However, these models are often expensive and cumbersome to implement [7]. As the number and quality of Internet models that provide self-directed psychotherapy [9], education [10], or social support [11] increases, so does the prospect for improving depression outcomes without costly person-to-person interventions or complex organizational changes [12]. Technology-based depression interventions have shown considerable promise in treating depression in select populations. However, the breadth of appeal of Internet-based treatment outside of highly structured surroundings, such as in school settings for children and adolescents, is not known [13-19].

Internet sites offering peer-to-peer interaction may be particularly attractive to laypersons. In Australia, peer-to-peer and gaming models are widely used, including Reach Out! which provides psycho-education regarding common adolescent mental health problems [20]. Similarly, in the United States, social media is commonly used to address health concerns. Approximately 28% of Internet users are estimated to have contacted an Internet support group (ISG) for a medical condition or personal problem [21]. ISGs have several advantages, including transcending geographic barriers, facilitating disclosure (people will often tell a computer more than to another person), and increasing access to diverse sources of information. There is some evidence that use of ISGs for depression may be associated with both reductions in depressed mood and an increase in learning about depression [11,22]. Although we know little about how to engage primary care patients with Internet-based models, it is theorized that social engagement will be an essential component [17,23]. Unfortunately, many Internet-based interventions have high dropout rates, ranging up to 75% of all participants [24]. Participants’ longer and more consequential involvement with the Internet site and its direct personal relevance to the user predict greater behavior change in Internet-based programs [25,26]. Peer support and frequent updates may support participation in ISGs [27,28]. A prerequisite for further evaluations of the potential benefits of ISGs for primary care patients with depressive illness is developing an effective method of referral and ascertaining key predictors of participation. Self-determination theory provides a framework for evaluating possible referral methods. In this model, internal source motivations based upon preferences for autonomy, competence, and connection are superior to external source motivations based upon financial incentives or recommendation of an authority figure [2].

The aim of this study was to determine the most feasible and effective methods to refer primary care patients with depressive disorders and symptoms to an ISG. We examined whether baseline attitudes based on the theories of motivational interviewing, planned behavior, and self-determination predicted Internet site participation [2,29], and we assessed whether a structured approach to goal setting would increase ISG participation [30]. We conducted a randomized controlled trial to compare 3 different methods of referring primary care patients with depressive disorders and symptoms to an ISG: emphasizing internal motivation (patient-oriented brochure), emphasizing external motivation (physician recommendation), and employing neither motivational strategy (referral card with Internet address). Interventions that require minimal physician involvement are particularly useful in actual practice settings. Because of this, the simple referral card is an important consideration. Our hypotheses were that (1) primary care referral (all methods) would be effective in engaging participants with the ISG (ie, >30% of participants would visit the ISG), (2) the physician letter recommendation (external motivation) group would show the greatest participation, (3) attitudes toward ISG participation would be important predictors of subsequent use, and (4) email reminders would increase participation.

## Methods

### Study Design

**Figure 1:**
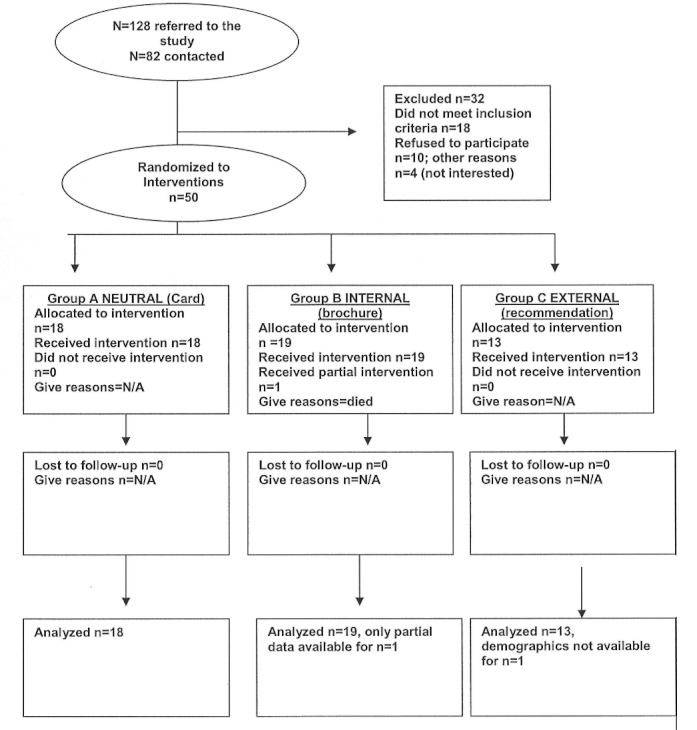
CONSORT diagram.

This study included 9 key steps from initial recruitment to study completion. We identified potential participants using 3 methods: (1) reading a poster in the waiting room and self-referred, (2) completion of a statement of interest form in their physician’s office after discussion with their physician, or (3) completing a statement of interest at a public information table in the primary care office. In step 2, those recruited through reading a poster and self-referred or completing a statement of interest form after discussion with their physician provided consent to be called by study staff to learn more about the study (those who signed up at the public information table in the primary care office already knew about the study). Step 3 was a phone eligibility assessment. After providing consent for phone eligibility assessment, the study coordinator conducted a brief structured psychiatric interview based on the Patient Health Questionnaire-9 (PHQ-9) [31]. Following eligibility confirmation by both the principal investigator (internist-pediatrician) and co-investigator (psychiatrist), participants were offered enrollment in the study (step 4). There was approximately a 7-day delay between initial eligibility assessment and actual enrollment. At step 5, the study coordinator met the participants at or near the primary care office and conducted informed consent and enrollment. At this time, the participants also completed a baseline written questionnaire. In step 6 after consent and enrollment, participants were randomly assigned (using a sealed envelope with equal likelihood of assignment to all arms) to one of 3 groups: (1) referral card (neutral motivation group), (2) patient-oriented brochure providing examples of online postings and experience (internal motivation group), or (3) physician letter of recommendation (external motivation group) (see Figure 1). Participation on the Internet was assessed via automated activity tracking (step 7). Study staff contacted each participant 6 to 8 weeks after enrollment to evaluate depressed mood and any concerns about their website experiences (step 8). In the final step (step 9), each participant was asked to complete a written poststudy questionnaire. The study was approved by the University of Chicago Institutional Review Board. The trial was prospectively registered (ClinicalTrials.gov NCT00886730).

### Inclusion and Exclusion Criteria

All eligible participants met the following criteria: (1) a PHQ-9 score of 8 or above with either depressed mood or anhedonia and/or were considering treatment for depressed mood, (2) were accepting of at least one form of treatment for depression, (3) had not viewed or posted messages more than once in the past month on any ISG, (4) did not self-report being diagnosed with bipolar disorder by a health professional, (5) was 18 years of age or older, (6) attends a primary care clinic and had visited it in the past 6 months, and (7) had Internet access for the subsequent 4 weeks, had been on the Internet at least 3 times, and was able to use email. We excluded those considered to be at high risk for suicide attempts, which included those with past psychiatric hospitalizations, past suicide attempts, diagnosed bipolar disorder, or a score of greater than 1 on the PHQ-9 suicide assessment item (bothered by thoughts that he/she would be better off dead or of hurting him/herself in some way more than half the days in past 2 weeks), or who reported intent for self-harm as per assessment.

### Setting

We recruited physicians at 6 primary care sites in urban and suburban areas of Chicago. These included a federally qualified health center (n=1), a private practice (n=1), university-affiliated off-site practices (n=2), a student health clinic (n=1), and an on-site academic resident practice (n=1). Physicians were recruited using lunchtime education programming.

### Adaptations Made After Study Initiation

Several barriers to implementation were addressed after the study was fielded. During the course of the study, 2 practice sites closed and several physicians left the practices. After initial attempts using nurses to approach patients were unsuccessful, physicians were asked to approach appropriate patients directly to determine if they might be interested in the study. The original intention was to have the recommendation letter be from the participant’s personal physician. Instead, the letter was signed by the principal investigator (a primary care physician) because of time feasibility limitations.

### Randomization, Blinding, and Concealment

We randomly assigned participants (using sealed envelopes) to 1 of the 3 groups described subsequently. A data manager who was not involved in the study execution prepared the sealed envelopes. Participants were aware of group assignments. The main outcome measures were collected in an automated fashion online. The database manager and the safety caller (described subsequently) were blinded to group assignments.

### Randomized Intervention Groups

#### Neutral Motivation Orientation (Referral Card)

Participants received a simple 3”×5” card with the name of the ISG and the following description: “Online support/coping group for people feeling blue, stressed, depressed, down, low, or sad” (see Multimedia Appendix 1 for full text).

#### Internal Motivation Orientation (Patient-Centered Brochure)

Participants received an 8”×11” handout that provided a more complete description of the ISG. The handout was based on a patient perspective with samples of Internet postings from current users. This card emphasized peer-to-peer support and did not mention health care organizations or health care provider endorsements. The information addressed potential barriers to participation: participants would not be identified, posting would not take much time, information from peers could be checked for accuracy with other peers and providers, and patients could learn how to tell their providers about their activities on the Internet (see Multimedia Appendix 1 for full text).

#### External Motivation Orientation (Physician Recommendation)

Participants received a medical recommendation in the form of a letter signed by the principal investigator, a primary care physician (see Multimedia Appendix 1 for full text).

#### Email Reminder

An email reminder was sent to all participants who did not visit the portal within 7 days of enrollment. Email reminders have been associated with higher levels of participation [27].

#### Internet Portal Intervention

We constructed a portal to connect primary care patients to both fact-based information and an established ISG. Two focus groups identified several key ideas with regard to the development of a primary care portal: (1) offer opportunities to learn from others, (2) offer opportunities to help others, (3) offer fact-based resources to evaluate accuracy of information, and (4) protect users from disturbing content, such as self-harm or salacious topics. To address these needs [32-34], we chose to build a portal (Psycho-Babble) that would: (1) offer access to fact-based information sites (eg, NIMH [35] and MOODGym [36])*,* (2) present aspects of the ISG that focus group participants suggested would be of greatest interest, and (3) offer access to the ISG. Participants could use the portal to visit the ISG and also the other websites. The ISG allowed participants to view messages and then decide if they actually wished to register there.

### Internet Support Group

**Figure 2:**
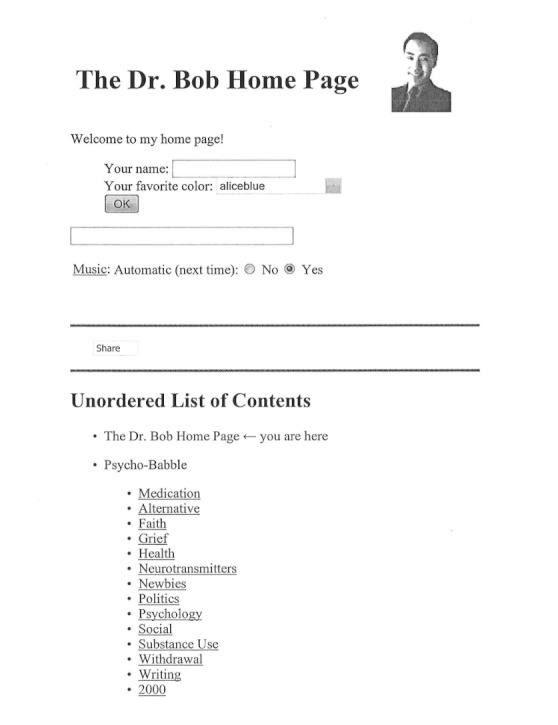
Screenshot of Psycho-Babble home page.

Psycho-Babble [37,38] is a mental health peer support group started in 1998 (see Figure 2). Online mental health groups can be classified as autonomous self-help groups or support groups led by mental health professionals. Psycho-Babble is a hybrid that combines the empowerment of self-help with the supportiveness of a milieu maintained by a mental health professional. The asynchronous online (message board) format makes the group more accessible and in some ways safer than groups that meet face-to-face [38]. The original forum continues to focus on biological treatments, and additional forums have been added for psychological treatments, complementary and alternative treatments, religious faith, social support, and discussion of the administration of the site. The portal through which participants reached Psycho-Babble highlighted 6 forums. The introduction in the main forum lists 3 other forums, and a link takes participants to a list of all 10. We expected participants to have been aware of the other Psycho-Babble forums. Higher user control has been associated with favorable perceptions by participants [39]. We believed Psycho-Babble would be a valuable ISG to use because of its well-developed participant base with history, loyalty, and repeat visits [40].

### Physician and Nurse Training

As part of this intervention, investigators met with each primary care provider and nurse at the participating primary care sites for 30 minutes during a lunchtime education program. This program included (1) explanation of the study design and the ISG and (2) summary of problems in depression care, self-management approaches currently used by individuals with depression, and the state of research on ISGs. A group of resident physicians received additional training on how to conduct randomized trials as an added inducement to participate in the study.

### Safety Monitoring

A structured approach to managing participant safety was used. After eligibility assessment, all participants with elevated depressed mood received a recommendation for a medical evaluation. All those with self-harm ideation received a structured assessment to determine need for service. While enrolled in the study, the existing Psycho-Babble adverse event reporting mechanisms were available to participants. A safety call was made by a licensed clinical social worker 6 to 8 weeks after enrollment [37,38].

### Outcome Measures

We evaluated use of the portal and the ISG by using data automatically logged by the portal and ISG servers (Table 1). We originally planned to assess outcomes across several categories: visits, time logged in, posts viewed, and posts posted. Few posts were posted; therefore, we added a broader measure of interaction, communications, which we use to refer to all submissions of data by participants. Participants submitted data not only for postings, but also to preview posts, to take the quiz required to register at the ISG, to enter a synchronous (real-time) chat room, to actually chat, and to contact the ISG administration.

#### Portal Measures

The portal server tracked visits and minutes logged in by each username. Participants were automatically logged out after 6 minutes. Time actually engaged with the portal may have been longer (eg, if they browsed the NIMH site for 30 minutes) or shorter (eg, if they stepped away from their computer after 3 minutes). If participants used the portal to visit the ISG, the time they spent at the ISG was included in the time they were logged in to the portal. However, if they visited the ISG directly, it was not. It was possible to access the ISG directly because it was open to the public and not restricted to study participants.

#### Internet Support Group Measures

At the ISG, viewing posts was unrestricted, but submitting data, including postings, was restricted to registered group members. The ISG server automatically tracked viewing of posts by participants by using cookies (information stored by a server on a user’s computer) which it set when participants visited the ISG from the portal. Because participants could visit the ISG directly or delete the cookies, viewing of posts (ie, ISG visits) by study participants may have been undercounted. The ISG server automatically tracked communications, including postings, by username.

### Independent Variables

#### Sociodemographic Factors

We obtained self-reported age (years), gender, race/ethnicity, and education level (eg, some high school or high school graduate).

#### Mood

For the participant’s dimensional measures of depressed mood, we used the 10-item Center for Epidemiologic Studies Depression Scale (CES-D) (Cronbach alpha =.87) [41] and the PHQ-9 [31] score (Cronbach alpha=.77).

#### Self-efficacy

Perceived control was assessed with the Mastery Scale [42]. The 7 items on the scale measure the extent to which participants see themselves as being in control of the forces that significantly affect their lives. Higher scores indicate greater self-efficacy (Cronbach alpha=.81).

#### Loneliness

The loneliness item of the CES-D was used to report loneliness. Respondents were asked the frequency of feeling lonely in the past week on a 4-point scale (0=less than a day, 1=1-2 days, 2=3-4 days, and 3=5-7 days).

#### Self-determination Theory

Self-determination theory posits that humans seek experiences in which they will develop autonomy, connection, and competence. Furthermore, the stronger the internal motivation (as opposed to external factors), the higher the quality and enduring the motivation [2]. Based on a focus group, we developed items to address attitudes in each of these domains and 1 additional one, concern for adverse experiences (see Multimedia Appendix 1). We measured 9 items based on a Likert-type scale ranging from 1 (strongly disagree) to 5 (strongly agree) (Cronbach alpha=.69).

#### Theory of Planned Behavior

We revised the items from a previous questionnaire, which was modified based on the preventive health model and the theory of planned behavior [43,44] for the purpose of participation in our ISG. According to the theory of planned behavior, intention is the most proximal cognitive measure to actual behavior. Intention is directly influenced by attitudes and beliefs toward a behavior (eg, attitudes toward intervention), subjective norms (eg, concerns with regard to family, peer, or employer opinions), and perceived behavioral control (ie, controllability and self-efficacy) [29]. We measured items based on a Likert-type scale (1=strongly disagree to 5=strongly agree) for each domain (see Multimedia Appendix 1). The reliability (Cronbach alpha=.89) and predictive validity of the original instrument has previously been demonstrated in adolescents [45].

#### Transtheoretical Model of Change

Items were adapted from Miller and Rollnick’s 3-item assessment of motivation [46]. In terms of validity, this scale and many individual items predicted adherence to an Internet-based depression prevention intervention (modified form) [45]. On a scale from 1 to 10, participants rated the importance, their ability, and their readiness to overcome depressed mood over the next 2 months. Higher numerical values indicate higher levels for agreement with the item.

#### Shared Decision Making

We previously demonstrated improved outcomes with shared decision making for depression [47]. We asked participants to rate agreement with “I can talk with my providers in a way so my preferences for treatment are included” on a Likert-type scale (1=strongly disagree to 5=strongly agree).

### Analysis

Descriptive statistics were calculated for the demographic variables. For between-group categorical comparisons, we used Pearson’s chi-square test or the Fisher exact test when there were <5 observations per cell. For continuous outcomes, we used analysis of variance (ANOVA) for between-group comparisons at the same time points. For continuous between-group data with skewed distribution, we used the Mann-Whitney test for 2-level comparisons. We used a similar analytical approach for comparisons between the 3 groups and also used the Kruskal-Wallis test for 3-level comparisons with a skewed distribution. We developed regression models for 3 main outcomes: (1) total time on-site, (2) number of posts viewed, and (3) number of posts attempted. Separate regression analyses were performed for the outcome variables. For total time on-site, the outcome had skewed data and there were values of zero, precluding a direct logarithmic transformation. Therefore, we added a value of 1 to all the variables so that they could be logarithmically transformed. Linear regression was then performed. For the count data (posts viewed and posts attempted), negative binomial regression was used. Because of the limited number of observations, we chose to develop only 2 models for each analysis. Model 1 was a univariate analysis. Model 2 adjusted for all items that were significant at *P*<.05 on Model 1 for each outcome. STATA 11 (StataCorp LP, College Station, TX, USA) was used to conduct the analyses. We also calculated effect sizes for between-group comparisons with statistical significance (Cohen’s *d*) [48].

### Sample Size Calculations and Interim Analyses

A sample size of 225 was estimated to have a power of 0.8 to detect the difference between both the neutral group and the internal or external group (50%, 35%, and 10% participation). This sample size was not achieved because of an extensive delay in starting the study due to an extensive Institutional Review Board review (1 year). An interim analysis was conducted for purposes of safety review and end of grant funding. On the basis of the results of the interim analysis, which showed significant differences between randomization groups for the main study endpoints and significant predictors for measures of participation, the data monitoring and safety board (DSMB) and the investigators jointly agreed to end the trial.

**Table 1:** Outcome variables.

Items		Description	Source^a^
**Sessions and time**			
	Number of sessions	Number of individual visits	Portal
	Percent those in sessions with at least 1 session	Percent with at least 1 visit	Portal
	Total time	Number of minutes spent logged in (participants were timed out after 6 minutes of inactivity)	Portal
	Minutes elapsed per session	Number of minutes/session	Portal
**Viewing**			
	Posts viewed	Number posts viewed by participant	ISG
	Percent who viewed at least once	Percent who viewed at least 1 post	ISG
	Posts viewed by each participant who registered	Number of posts viewed by those who registered on the ISG	ISG
	Boards viewed	Number of boards viewed per participant	ISG
	Boards viewed by each participant who registered	Number of boards viewed per participant who registered with the ISG	ISG
**Posting**			
	Communications	Posts and data combined	ISG
	Data submitted	Information submitted to the portal	ISG
	Posts submitted	Number of posts submitted	ISG
	Posts attempted	Number of posts attempted	ISG
	Percent of those who attempted at least once	Percent who attempted to post at least 1 time	ISG
	Posts attempted by each participant who registered	Number of posts submitted who registered on the ISG	ISG
	Posts posted	Number of posts attempted who registered on the ISG	ISG
	Percent of those who posted at least once	Percent who attempted to post at least 1 time who registered on the ISG (created post, but failed to confirm)	ISG
	Posts posted by each participant who registered	Mean number of posts submitted who registered on the ISG	ISG
**Visitation**			
	Visited portal or ISG	Percent who visited either the portal or directly went to the ISG	ISG and portal
	Participants who visited portal	Percent who visited the portal	ISG and portal
	Participants who registered ISG	Percent who registered on the ISG	ISG and portal

^a^ ISG: Internet support group.

## Results

### Sample Characteristics

A total of 50 participants were assigned to the 3 groups. At baseline, there were no significant differences between the 3 groups on any demographic characteristics (Table 2). With regard to the race/ethnicity of the sample, 47% were white (23/49), 41% were African American (20/49), 2% were Hispanic (1/49), 6% were Asian (3/49), and 4% were other (2/49). One participant did not report race/ethnicity. The mean age of participants was 37.49 years (SD 17.15). This was a moderately depressed cohort with PHQ-9 scores ranging between 7.22 to 10.24 for the 3 randomization groups with a mean for the entire sample of 9.18 (SD 4.64). With regard to education and socioeconomic status, 41% (20/48) were college graduates and the mean reported income was US $45,407 (SD 59,496). Fifty-one percent (25/49) reported discussing depression with their physician, 43% (21/49) reported being treated before for depression, and 31% (15/49) reported a family member had been depressed in the past. One participant did not provide any demographic data. Over the course of the study (November 2008 to June 2009), 128 individuals were referred by their physicians or answered advertisements posted in the clinics, of which 82 were successfully contacted and had eligibility assessed with 32 being excluded (see Multimedia Appendix 1). Reasons for exclusion included 10 who were eligible but did not enroll, 4 who were not interested, and 18 who did not meet eligibility criteria (8 with past psychiatric hospitalization, 8 with a self-harm attempt, and 2 with bipolar disorder). Of those who could be reached (82), only 4 reported no interest in the study. Primary care physicians recruiting for the study did not report many refusals for initial contact by study staff. Of those referred to the study staff for contact, 64% were contacted. Partial data were available for 2 participants, 1 who died of natural causes after enrollment (internal motivation group) and 1 participant enrolled in the external motivation group. 

### Participation for Entire Cohort

A total of 31 of 50 participants (62% of the entire study cohort) visited the portal or the ISG directly, whereas 27 of 50 (54%) registered on the ISG itself. Participants who visited the portal logged in for a mean of approximately 2 hours (118.5 minutes) each. Participants who visited the ISG viewed an average of 20.9 posts, communicated an average of 5.8 times, and posted an average of 0.4 posts. Table 3 shows the frequency distributions of the number of visits to the portal, time on the portal, and the number of posts viewed, communications, and posts posted. There was a skewed distribution spent on time on portal with 20 (40%) having no time, but 1 (2%) with 1301 minutes. Similarly, 4 (8%) participants viewed more than 46 posts, whereas 25 (50%) viewed no posts. In terms of communications, most (40/50, 80%) made no communications whereas a few (3/50, 6%) communicated more than 15 times. In terms of actual postings, 47 (94%) never posted, whereas 1 posted 1, 4, and 6 times. Most posts and replies were not replied to (new posts: 3 replied to, 6 not replied to).

### Participation by Randomization Group

The internal motivation group had significantly greater participation for several participation measures than the neutral motivation group with more individual data submissions (mean 7.53 vs mean 0.39, *P*=.01, effect size [ES] 0.67, CI –0.02 to 1.32), were more likely to attempt to submit at least once (43% vs 6%, *P*=.02) and had significantly more posts attempted overall and by those who registered (mean 5.21 vs mean 0.17, *P*=.02, ES 0.62, CI –0.07 to 1.27 and mean 0.19 vs mean 0.01, *P*=.02, ES 0.55, CI –0.19 to 1.26, respectively). The neutral motivation group spent significantly more time logged onto the ISG as compared to the external motivation group (mean 9.27 vs mean 0.23, *P*=.04, ES 0.44, CI –0.29 to 1.15). There was only one 3-group comparison (see Multimedia Appendix 1) that demonstrated significance, which was the percent posting at least once (*P*=.02). The internal motivation group had significantly greater participation for several participation measures than the external motivation group (Tables 4 and 5). Specifically, members of the internal motivation group had more individual data submissions (mean 7.53 vs mean 0.54, *P*=.03, ES 0.60, CI –0.14 to 1.31), were more likely to submit at least once (42% vs 8%, *P*=.05), were more likely to have visited with the portal or ISG directly (79% vs 46%, *P*=.05) and were more likely to have attempted to post overall and by those who registered (mean 5.21 vs mean 0.23, *P*=.05, ES 0.56, CI –0.18 to 1.27 and mean 0.19 vs mean 0.01, *P*=.05, ES 0.55, CI –0.19 to 1.26, respectively). No other significant differences were observed.

### Email Reminders

During the course of the study, 22 participants received an email reminder because they had not visited the portal within 7 days of enrollment. Of these 22 participants, 5 subsequently visited the portal; of those 5 participants, 3 did so within 2 days of receiving the email reminder.

### Predictors of Participation

#### Time On-Site

In the univariate analysis, demographic characteristics, severity of depressed mood, and self-efficacy did not predict minutes logged in (Table 6). One attitude item, “might read troubling comments,” was a statistically significant negative predictor (*P*=.03). Three items, “participating is important,” “want to do what physicians want me to do,” and “going to a depression Internet support website is easy,” were statistically significant (*P*=.049, *P*=.02, and *P*=.047, respectively), and another 3, “benefits outweigh difficulty,” “want to do what family thinks,” and “physicians think I should go” were not statistically significant (*P*=.06, *P*=.08, and *P*=.07, respectively). The motivation items and participation in decision making were not statistically significant. In the exploratory multivariate analysis of the significant univariate predictors, only the item of “might read troubling comments” remained statistically significant (*P*=.03).

#### Posts Viewed

Demographic characteristics and mood did not predict posts viewed (Table 7). Self-efficacy was not statistically significant (*P*=.09). No attitude items were statistically significant. Two intention items were statistically significant (“friends think I should go” *P*=.03, and “physicians think I should go” *P*=.01) and 1 was not statically significant (“want to do what friends think” *P*=.08). The motivation items and participation in decision making were not statistically significant. In the exploratory multivariate analysis of the significant univariate predictors, none remained statistically significant.

#### Posts Attempted

Demographic characteristics did not predict posts attempted (Table 8). Baseline CES-D, was not statically significant (*P*=.06). Self-efficacy was statistically significant (*P*=.04). Two intention items, “my family supports me going” and “physicians think I should go,” were statistically significant (*P*=.04 and *P*=.046, respectively), and another 2, “friends think I should go” and “want to do what physicians think,” were not statically significant (*P*=.06 and *P*=.06). Participation in decision making was not statistically significant. In the exploratory multivariate analysis of the significant univariate predictors, only self-efficacy remained statistically significant (*P*=.05).

### Safety and Unintended Events

Fourteen of 49 participants received follow-up calls from the principal investigator related to concerns identified by the assessment caller (ie, depressed mood or self-harm ideation). One participant died of natural causes shortly after enrollment. No study participant reported adverse experiences while on the ISG and there were no self-harm events.

**Table 2:** Demographics of study participants.

Items		Participants (N=50)			*P* value
		Neutral motivation (card) n=18	Internal motivation (brochure) n=19	External motivation (recommendation) n=13	
Age (years), mean (SD)		34.83 (12.72)	39.39 (16.83)	34.42 (19.49)	.62
PHQ-9 score, mean (SD)		9.13 (5.84)	10.24 (3.72)	7.22 (3.83)	.29
**Gender, n (%)**					
	Male	4 (22)	6 (33)	5 (42)	.54
	Female	14 (78)	12 (67)	7 (58)	
**Ethnicity, n (%)**					
	White	10 (56)	8 (44)	5 (42)	.71
	Non-white	8 (44)	10 (56)	7 (58)	
**Education, n (%)**					
	Some high school	3 (17)	1 (6)	2 (17)	.72
	High school graduate	1 (6)	2 (11)	2 (17)	
	Some college	5 (28)	7 (39)	5 (42)	
	College graduate	9 (50)	8 (44)	3 (25)	
**Marital status, n (%)** ^**a**^					
	Married	1 (6)	1 (24)	1 (8)	.62
	Divorced/separated/widowed	2 (11)	2 (12)	1 (8)	
	Never married	3 (15)	11 (65)	10 (83)	
**Talk to health care provider, n (%)**					
	Yes	11 (61)	10 (59)	3 (27)	.19
**Treatment, n (%)**					
	Yes	8 (57)	9 (69)	4 (50)	.75
**Family treatment, n (%)**					
	Yes	5 (28)	8 (47)	2 (17)	.22
**Counseling, n (%)**					
	Yes	10 (56)	12 (71)	6 (50)	.56
Income (US$), mean (SD)		35,025.00 (30,344.51)	57,553.67 (84,277.86)	43,056.67 (52,325.73)	.90

^a^ Marital status does not equal the total sample size in each group because data were not obtained from some participants.

**Table 3:** Time on portal, posts viewed, and number of communications for entire cohort.

Items		Participants, n (%)^a^
**Time on portal (minutes)** ^**b**^		
	0	20 (40)
	1	6 (12)
	2	2 (4)
	3	2 (4)
	4	3 (6)
	5	2 (4)
	6	1 (2)
	9	1 (2)
	13	1 (2)
	15	1 (2)
	16	1 (2)
	19	1 (2)
	30	1 (2)
	31	1 (2)
	45	1 (2)
	51	1 (2)
	53	1 (2)
	89	1 (2)
	543	1 (2)
	774	1 (2)
	1301	1 (2)
**Number of posts viewed**		
	0	25 (50)
	1	1 (2)
	2	4 (8)
	3	2 (4)
	4	2 (4)
	5	1 (2)
	6	1 (2)
	9	2 (4)
	12	1 (2)
	15	2 (4)
	16	1 (2)
	17	2 (4)
	20	1 (2)
	46	1 (2)
	54	1 (2)
	55	1 (2)
	92	1 (2)
	152	1 (2)
**Number of communications**		
	0	40 (80)
	3	1 (2)
	4	1 (2)
	7	3 (6)
	8	1 (2)
	12	1 (2)
	16	1 (2)
	34	1 (2)
	59	1 (2)

^a^ For simplicity, all those enrolled in the study but who did not visit the site are recorded as 0.

^b^ The time in minutes is the actual time each participant spent logged in to the portal.

**Table 4:** Participation measures reporting only with *P*<.10.^a^

Items		Neutral motivation (card) n=18	Internal motivation (brochure) n=19	External motivation (recommendation) n=13
**Time, mean (SD)**				
	Total time		74.68 (228.13)	4.5 (8.63)
	Minutes logged (portal)		56.21 (175.66)	4.08 (8.3)
	Minutes logged (ISG)	9.27 (26.59)	18.47 (53.24)	0.23 (0.6)
**Posting**				
	Data submitted, mean (SD)	0.39 (1.65)	7.53 (15.09)	0.54 (1.94)
	Post attempted, mean (SD)	0.17 (0.71)	5.21 (11.54)	0.23 (0.83)
	Percent of those who attempted to post at least once, n (%)	1 (6)	2 (42)	1 (8)
	Post attempted by each participant who registered, mean (SD)	0.01 (0.03)	0.19 (0.43)	0.01 (0.03)
**Portal and ISG, mean (%)**				
	Visited portal or ISG		15 (78.95)	6 (46.15)

^a^ For full data, see Multimedia Appendix 1.

**Table 5:** Group comparisons of participation measures reporting only with *P*<.10.^a^

Items		2-group *P* value		
		Neutral (card) vs internal (brochure)	Neutral (card) vs external (recommendation)	Internal (brochure) vs external (recommendation)
**Time**				
	Total time			.08
	Minutes logged (portal)			.09
	Minutes logged (ISG)		.04	.08
**Posting**				
	Data submitted	.01		.03
	Post attempted	.02		.05
	Percent of those who attempted at least once	.02		.05
	Post attempted by each participant who registered	.02		.05
**Portal and ISG**				
	Visited portal or registered ISG			.05

^a^ For full data, see Multimedia Appendix 1.

**Table 6:** Predictors of time on-site reporting only with *P*<.10.^a^

Predictors		n	Mean (SD)	Model 1			Model 2		
				Beta	SE	*P*	Beta	SE	*P*	
**Attitudes toward Internet including self-determination theory**									
**Concern for adverse experiences**									
	I might read troubling comments about depression on the Internet	48	3.67 (1.12)	-0.58	0.25	.03	-0.62	0.28	.03
**Preventive health model questions**									
**Beliefs about intervention**									
	Participating in a depression Internet social support website is an important thing to do	46	3.59 (1.02)	0.58	0.29	.049	0.27	0.32	.40
**Attitudes toward intervention**									
	The benefits of a depression Internet social support site outweigh any difficulty	46	3.48 (0.86)	0.66	0.34	.06			
**Social norms**									
	I want to do what the members of my immediate family think I should do	43	2.98 (1.03)	0.54	0.30	.08			
	Physicians and other health care professionals want me to go to an Internet intervention site	45	3.02 (1.2)	0.47	0.25	.07			
	I want to do what the physicians and other health care professionals want me to do	45	3.4 (0.91)	0.8	0.32	.02	0.45	0.36	.23
**Self-efficacy**									
	Visiting this depression Internet social support site is an easy thing to do	46	3.89 (0.97)	0.62	0.30	.047	0.43	0.29	.16

^a^ For full data, see Multimedia Appendix 1.

**Table 7:** Predictors of posts viewed reporting only with *P*<.10.^a^

Predictors		n	Mean (SD)	Model 1			Model 2		
				Beta	SE	*P*	Beta	SE	*P*
**Social factors**									
	Self-efficacy scale	45	0.44 (0.87)	0.63	0.37	.09			
	Preventive Health Model								
**Social norms**									
	My close friends support me going to depression social support Internet site	45	2.78 (1.13)	0.85	0.4	.03	0.13	0.55	.81
	I want to do what my close friends think I should do	45	2.82 (0.98)	0.93	0.53	.08			
	Physicians and other health care professionals want me to go to an Internet intervention site	45	3.02 (1.20)	0.88	0.33	.01	0.79	0.49	.11

^a^ For full data, see Multimedia Appendix 1.

**Table 8:** Predictors of posts attempted reporting only those with *P*<.10.^a^

Predictors		n	Mean (SD)	Model 1			Model 2		
				Beta	SE	*P*	Beta	SE	*P*
**Mood**									
	Baseline CES-D	43	9.19 (4.64)	0.28	0.15	.06			
	Social factors								
	Self-efficacy scale	45	0.44 (0.87)	1.56	0.76	.04	1.5	0.77	.05
**Attitudes toward Internet including self-determination theory**									
**Connection seeking**									
	Able to help others by sharing my experiences on the Internet	47	3.53 (1.08)	2.05	1.07	.06			
**Preventive health model**									
**Social norms**									
	My close friends support me going to depression social support Internet site	45	2.78 (1.13)	2.04	1.07	.06			
	My immediate family supports me going to a depression Internet social support site	45	2.71 (1.25)	1.58	0.75	.04	1.07	0.57	.06
	Physicians and other health care professionals want me to go to an Internet intervention site	45	3.02 (1.2)	0.81	0.41	.046	0.23	0.43	.60
	I want to do what the physicians and other health care professionals want me to do	45	3.4 (0.91)	1.28	0.67	.06			
**Transtheoretical model of change/MI**									
	Readiness	48	7.81 (2.73)	0.65	0.36	.07			

^a^ For full data, see Multimedia Appendix 1.

## Discussion

This study suggests that low-cost and relatively low-intensity primary care referral methods are useful for engaging ISG-naive patients with depressed mood with a depression ISG. Our results support hypothesis 1 that referral of primary care patients to an Internet-based social support group portal was effective (>30% visited the site in all groups). Hypothesis 2 was not supported, whereby a patient-oriented brochure primarily focused on eliciting internal motivations demonstrated greater participation on multiple measures with moderate effect sizes than a generic medical letter of recommendation. Support for hypothesis 3 was attained with the findings that items from several models, but particularly the theory of planned behavior (in particular social norms), predicted participation. Furthermore, there was some evidence that email reminders to eligible patients were associated with the first visit to the portal (hypothesis 4).

This is the first trial that we are aware of comparing low-intensity methods for referring primary care patients with depression to online social support or other online depression resources. The percentage of patients visiting the portal (79%) in the internal motivation (brochure) group was similar to those in other studies using minimal contact approaches in medical settings of brief advice (85%) and email reminder (77%) [49,50] versus participation in a mental health information website without any referral method in the general population (24%) [10]. We previously demonstrated that more intensive interventions (motivational interviewing versus brief advice) increases participation for Internet-based depression prevention interventions for adolescents, with similar moderate effect sizes [50]. Others have demonstrated that email-based reminders may increase participation [49]. Resnicow [51] demonstrated that health messaging using brochures that relate closely to the patients’ current concerns and preferred method of motivation may significantly increase prohealth behaviors such as diet change. The preparation of our brochure based on themes identified in 2 focus groups may have been particularly helpful. Similarly, relying on the brochure may have enabled our participants to develop higher quality intrinsic motivations as opposed to external ones. It is noteworthy that the greatest impact of the referral methods was on measures of posting, rather than simply viewing, which perhaps supports the premise that higher quality motivation is critical to enacting higher levels of engagement. Goal setting and having an established motivational structure to bring to the Internet experience has been noted to influence participation and could explain the added benefit of the intrinsic group (brochure) as well [30]. Similarly, perhaps the letter (external motivation) tended to crowd out intrinsic motivations in favor of external ones and serve as a negative incentive [52]. The finding that email reminders may increase participation is consistent with the findings of a prior systematic review [20]. However, it should be noted that the level of active involvement, such as posting, was very low (only 4 individuals posted).

Attitudes and clinical factors, rather than demographic factors, provided a broader understanding of the process by which participants may choose to participate in our mental health ISG. Typically, factors that predict participation in a self-directed psychotherapy website include demographic factors (younger age, higher education, and greater illness severity), clinical factors (greater dysfunctional thinking and illness severity), health services factors (referral by a mental health professional), and attitudes (importance, self-efficacy, and perceived benefits) [50,53,54]. Although the attitudinal predictors of participation in an ISG are similar to those for Internet-based self-directed psychotherapy, social norms appear to be more important factors in predicting participation in an ISG [44,50,55-57]. Perhaps participants considered using an Internet-discussion group to be more of a social choice than a treatment decision or event, and when considering something unfamiliar they defer to the judgment of others. It is uncertain how much participation on an ISG could be as sensitive to comparisons of activity with other individuals such as that occurs with gaming on the Internet [28].

These sites reflected the challenges of contemporary primary care, as 2 of the practice sites closed during the study and were relocated as a result of the financial crisis of 2008. In terms of internal validity, the participants were effectively randomized with no significant between-group differences and received 3 distinct interventions. Although small, the sample size demonstrated between-group differences and helped illuminate several preliminary associations. However, several key limitations with internal validity should be recognized: (1) the sample was quite small and the smallest group had only 13 participants (we only analyzed 12), (2) a letter from the patient’s own physician personal letter of recommendation might have performed better than a generic one, (3) the closure of 2 clinics during the study disrupted recruiting and in some cases delivery of the intervention elements, (4) delivery of emails to all groups and direct physician recruitment of nearly all participants may have resulted in reduced between-group differences in participation, and (5) poor engagement may simply have reflected lack of easy access to the Internet or experience with interfaces like those of the portal and ISG. The observation that the external motivation (recommendation) group assignment had a mean PHQ-9 of 7.22, which was below the eligibility criteria, suggests this group had disproportionate improvement in depressed mood before actual enrollment. Two forms of bias may explain this: (1) practice effect bias suggests that if the same test is administered within 2 weeks of time then practice effect bias is present (ie, a person remembers the questions and might try to change the answers), or (2) experimenter bias (participants may have guessed we were expecting to find decreased depression and answered to satisfy us). This could suggest that this group varied from the others in some unmeasured characteristic, such as motivation, but this seems unlikely to alter the result.

In terms of external validity, we should note several concerns as well. Although it is possible that some participants were not ISG-naive, the exclusion of only those who had not visited a depression ISG within the past 6 months is unlikely to have resulted in recruitment of participants who were significantly more disposed to depression ISGs than the general population. Because we did not collect data on prior use of other depression ISGs, we cannot determine the extent to which prior use may have influenced participation in the ISG of this study. Similarly, the recruiting methods may have self-selected for more motivated individuals, a fact that may suggest caution in estimating future effectiveness in primary care. However, recruiting physicians did not report many patients refusing referrals to the study and 64% of those referred were contacted. Given that this was perhaps a somewhat motivated sample, just half of the participants actually registered on the ISG. Lack of Internet access in this relatively lower income population and a somewhat outdated interface may have reduced participation versus a population with greater Internet access and skill [58]. Similarly, the degree to which the website may not have met current expectations for frequently updated materials, such as the style of the site, may have affected participation [58,59].

In conclusion, primary care patients may be amenable to referral to Internet-based social support and information portals, even if they have not utilized ISGs in the past. Relatively low-cost materials and minimal physician interaction may be required to accomplish this referral process. Motivational approaches focused on intrinsic factors may be superior to extrinsic ones. Referral to Internet-based social support using relatively low-cost methods, such as brochures, may offer a key method to moving toward patient-centered models, particularly for an illness in which treatment adherence and perhaps even outcomes are substantially influenced by prevailing social norms and attitudes. Clinicians may wish to consider referral to such reliable and reputable Internet-based social media as an early step to enable patients to explore with others treatment options or barriers to adherence. Health administrators and health services researchers should consider streamlined approaches to engaging patients with reliable and reputable Internet-based supportive social media. The next logical step would be a randomized clinical trial in a primary care setting to determine if referral to Internet-based social support for those with depressive illness improves quality of care, symptoms, and function.

## Multimedia Appendix 1

Group assignment samples and extended analysis of the tables embedded in the body of the manuscript.

## Multimedia Appendix 2

CONSORT eHealth Checklist V1.6.2 [36].

## Abbreviations

ANOVA: analysis of variance
CES-D: Center for Epidemiologic Studies Depression Scale
ES: effect size
ISG: Internet support group
PHQ-9: Patient Health Questionnaire-9
