# Supplementary material for: Internal Versus External Motivation in Referral of Primary Care Patients with Depression to an Internet Support Group: Randomized Controlled Trial
Source: J Med Internet Res. 2013 Mar 12;15(3):e42. doi: 10.2196/jmir.2197 (PMC3636270; doi:10.2196/jmir.2197)
Supplement: Supplementary file 1 [file jmir_v15i3e42_app1.pdf]

Multimedia Appendix 1

Figure 1. Neutral Motivation Group Card

Psycho-Babble

[URL]

Online support/coping group for people feeling blue, stressed, depressed, down, low, or sad

Figure 2. Internal Motivation Group Brochure

Psycho-Babble
[URL]
A website to help individuals with depression recover.

What is Psycho-Babble?

Psycho-Babble is an online community for people feeling blue, depressed, stressed, down, low, or sad and want to get ideas and information from other people with similar experiences. It is anonymous and interactive; discussion is led by Psycho-Babble users. The site is moderated by a professional in the field of mental health for civility. The site is not monitored for accuracy of information.

Psycho-Babble has already helped hundreds of people find information and support about depression online. Psycho-Babble was created by Dr. Bob Hsiung who is a professor at the University of Chicago. Psycho-Babble includes depression information and support. You can post questions or comments to other users on Psycho-Babble's dozens of message boards or ask Dr. Bob questions. The website includes sections for discussion on medication, therapy, alternative treatments, diet and nutrition, and even politics. You can come to learn, help others, or to just be social. The Psycho-Babble Internet site is not affiliated with the University of Chicago. Johns Hopkins University and the University of Chicago have received a grant from the National Institutes of Health to study methods of referring primary care patients to Internet based social support sites.

If you have any questions about this study, feel free to email Dr. Benjamin Van Voorhees at the University of Chicago. He is the principal investigator of this study. His email address is: [email address]

The discussion at Psycho-Babble is led by Psycho-Babble users, so you can talk about nearly anything. Here are some of the features offered on the Psycho-Babble website:

- Interactive message board moderated by a physician to discuss treatments, medications and therapy with other Psycho-Babble users
- Information on books, website and other resources for learning about psychotherapy and biological treatments

- Social support network for discussing a wide range of issues like faith, health and wellness, grief and mourning and alternative treatments.

Read what some users have said about the site

"This board helps me feel not alone, which is so important to me. If that is the only purpose it serves, well, that sometimes is more helpful to me than a lot of the meds."

"The board helps a lot in that I can feel a little connected to people who are also suffering, people who don't know me, won't judge me, and who have some darn good advice. After my long battles with mental illness and all the meds I've been on, I feel that I have something of value to offer, and it makes me feel good to pass it on."

Check out Psycho-Babble today! It's easy, it's fast, and you may just get an answer to that one difficult question or make a few friends.

Your personal username and password are: [username, password]

Once you log on you will be directed to create your own password

Figure 3. External Motivation Group Letter

RE: Internet Depression Support Group Study

Dear [participant name],

I am writing you to thank you for participating in the study "Feasibility Study of Methods of Referral Primary Care Patients with Depression for Internet-Based Social Support".

I want to encourage you to visit the Psycho-Babble Internet site. I believe you may find the experience of visiting the internet site helpful for you. This Internet site has been designed to be an online support group for people feeling blue, depressed, stressed, down, low, or sad. We hope that you will find information that will help you determine if and what form of treatment you need. Similarly, we hope you find the stories and comments of others who have had similar problems to be encouraging and helpful. Additionally, your comments may be helpful to others.

Please feel free to talk to me or the study staff or the study principal investigator, Dr. Benjamin W. Van Voorhees if you have any questions. The internet site address is: [URL].

Sincerely,

[doctor name], MD

Table 1. Randomization Groups and Participation

	Neutral- Card (A)			Internal -Brochure (B)				
	N	Mean/ (Percent)	SD (N)	*N*	Mean/ (Percent)	SD (N)	*2-group p-value*	*3-group p-value*
**Sessions**								
Number of sessions	*18*	1.33	2.11	*19*	2.95	5.92	.26	.33
Percent of those with at least one session	*18*	0.04	0.07	*19*	0.10	0.19	.26	.33
**Time**								
Total Time	*18*	115.44	333.36	*19*	74.68	228.13	.25	.17
Minutes logged (Portal)	*18*	106.22	324.07	*19*	56.21	175.66	.26	.24
Minutes logged (ISG)	*18*	9.27	26.59	*19*	18.47	53.24	.92	.20
Minutes per each participant	*18*	0.05	0.07	*19*	0.07	0.07	.25	.17
**Time & Sessions**								
Minutes per session	*18*	20.86	61.83	*18*	7.61	11.82	.40	.31
**Viewing**								
Post viewed	*18*	5.61	11.75	*19*	21.58	40.40	.21	.49
Percent who viewed at least once	*18*	(44.44)	(8)	*19*	(587.89)	(11)	.41	.68
Post viewed by each participant who registered	*18*	0.21	0.44	*19*	0.80	1.50	.21	.49
Boards viewed	*18*	1.11	1.68	*19*	1.53	1.87	.36	.52
Boards viewed by each participant who registered	*18*	0.04	0.06	*19*	0.06	0.07	.36	.52
**Posting**								
Data Submitted	*18*	0.39	1.65	*19*	7.53	15.09	**.01**	.10
Post attempted	*18*	0.17	0.71	*19*	5.21	11.54	**.02**	.17
Percent of those who attempted at least once	*18*	(65.56)	(1)	*19*	(42.11)	(2)	**.02**	**.02**
Post attempted by each participant who registered	*18*	0.01	0.03	*19*	0.19	0.43	**.02**	.17
Post posted	*18*	0.33	1.41	*19*	0.26	0.93	.63	.89
Percent of those who posted at least once (ISG)	*18*	(65.56)	(1)	*19*	(110.53)	(2)	1.00	.77
Posts posted by each participant (ISG)	*18*	0.01	0.05	*19*	0.01	0.03	.63	.89
**Visitation of Portal & ISG**								
Visited Portal or ISG	*18*	(565.56)	(10)	*19*	(798.95)	(15)	.13	.13
Participants who visited Portal	*18*	(565.56)	(10)	*19*	(743.68)	(14)	.25	.26
Participants who registered ISG	*18*	(565.56)	(10)	*19*	(587.89)	(11)	.89	.80
	Neutral Card (A)			External Recommendation (C)				
	N	Mean/ (Percent)	SD (N)	*N*	Mean/ (Percent)	SD (N)	*2-group p-value*	

**Sessions**								
Number of sessions	*18*	1.33	2.11	*13*	0.92	1.44	.65	
Percent of those in sessions	*18*	0.04	0.07	*13*	0.03	0.05	.65	
**Time**								
Total Time	*18*	115.44	333.36	*13*	4.50	8.63	.51	
Minutes logged (Portal)	*18*	106.22	324.07	*13*	4.08	8.30	.51	
Minutes logged (ISG)	*18*	9.27	26.59	*13*	0.23	0.60	**.04**	
Minutes per each participant	*18*	0.05	0.07	*13*	0.03	0.04	.41	
**Time & Sessions**								
Minutes used per session	*18*	20.86	61.83	*13*	3.75	8.37	.48	
**Viewing**								
Post viewed	*18*	5.61	11.75	*13*	4.00	5.74	.60	
Percent who viewed at least once	*18*	(44.44)	(8)	*13*	(46.15)	(6)	.93	
Post viewed by each participant who registered	*18*	0.21	0.44	*13*	0.15	0.21	.60	
Boards viewed	*18*	1.11	1.68	*13*	0.69	0.85	.76	
Boards viewed by each participant who registered	*18*	0.04	0.06	*13*	0.03	0.03	.76	
**Posting**								
Data Submitted	*18*	0.39	1.65	*13*	0.54	1.94	.81	
Post attempted	*18*	0.17	0.71	*13*	0.23	0.83	.81	
Percent of those who attempted at least once	*18*	(65.56)	(1)	*13*	(87.69)	(1)	1.00	
Post attempted by each participant who registered	*18*	0.01	0.03	*13*	0.01	0.03	.81	
Post posted	*18*	0.33	1.41	*13*	0.00	0.00	.40	
Percent of those who posted at least once (ISG)	*18*	(65.56)	(1)	*13*	(0.00)	(0)	1.00	
Posts posted by each participant (ISG)	*18*	0.01	0.05	*13*	0.00	0.00	.40	
**Portal & ISG**								
Visited Portal or Registered ISG	*18*	(55.566)	(10)	*13*	(46.156)	(6)	.61	
Participants who visited Portal	*18*	(55.566)	(10)	*13*	(46.156)	(6)	.61	
Participants who registered ISG	*18*	(55.566)	(10)	*13*	(46.156)	(6)	.61	

	**External Recommendation (C)**			**Internal -Brochure** (B)				
		Mean/	SD		Mean/	SD	*2-group*	
	N	(Percent)	(N)	*N*	(Percent)	(N)	*p-value*	
**Sessions**								
Number of sessions	*13*	0.92	1.44	*19*	2.95	5.92	.15	
Percent of those in sessions	*13*	0.03	0.05	*19*	0.10	0.19	.15	
**Time**								
Total Time	*13*	4.50	8.63	*19*	74.68	228.13	*.08*	
Minutes logged (Portal)	*13*	4.08	8.30	*19*	56.21	175.66	.09	

Minutes logged (ISG)	*13*	0.23	0.60	*19*	18.47	53.24	.08	
Minutes per each participant	*13*	0.03	0.04	*19*	0.07	0.07	*.054*	
**Time & Sessions**								
Minutes used per session	*13*	3.75	8.37	*19*	7.61	11.82	.11	
**Viewing**								
Post viewed	*13*	4.00	5.74	*19*	21.58	40.40	.50	
Percent who viewed at least once	*13*	(46.156)	(6)	*19*	(57.898)	(11)	.51	
Post viewed by each participant who registered	*13*	0.15	0.21	*19*	0.80	1.50	.50	
Boards viewed	*13*	0.69	0.85	*19*	1.53	1.87	.27	
Boards viewed by each participant who registered	*13*	0.03	0.03	*19*	0.06	0.07	.27	
**Posting**								
Data Submitted	*13*	0.54	1.94	*19*	7.53	15.09	**.03**	
Post attempted	*13*	0.23	0.83	*19*	5.21	11.54	*.052*	
Percent of those who attempted at least once	*13*	(7.698)	(1)	*19*	(42.118)	(2)	*.050*	
Post attempted by each participant who registered	*13*	0.01	0.03	*19*	0.19	0.43	*.052*	
Post posted	*13*	0.00	0.00	*19*	0.26	0.93	.23	
Percent of those who posted at least once	*13*	(0.001)	(0)	*19*	(10.531)	(2)	.50	
Posts posted by each participant who registered	*13*	0.00	0.00	*19*	0.01	0.03	.23	
**Portal & ISG**								
Visited Portal or Registered ISG	*13*	(46.151)	(6)	*19*	(78.959)	(15)	*.055*	
Participants who visited Portal	*13*	(46.151)	(6)	*18*	(73.684)	(14)	.11	
Participants who registered ISG	*13*	(46.151)	(6)	*19*	(57.898)	(11)	.51	

Table 2. Predictors of Time on Site

				**Model 1**		**Model 2**			
**Predictors**	*N*	*Mean/ (Percent)*	*SD*	*Beta*	*SE*	*P-value*	*Beta*	*SE*	*P-value*
**Mood**									
Baseline PHQ-9	48	13.07	4.44	-0.01	0.07	.92			
Baseline CESD 10	43	9.19	4.64	0.05	0.06	.40			
**Demographics**									
Age	48	36.44	15.98	0.01	0.02	.65			
Male	48	(31.25)	(15)	0.22	0.63	.73			
College Graduates*	48	(41.672)	(40)	0.34	0.59	.58			
Income	33	45407.70	59496.98	-1.11E-06	6.69E-06	.87			
**Social factors**									

Loneliness	34	1.18	0.97	-0.30	0.33	.37			
Self-efficacy scale	45	0.44	0.87	0.49	0.35	.17			
**Attitudes toward Internet including Self-Determination Theory**									
**Autonomy seeking**									
My identity to be anonymous while I am exploring concerns on the Internet	47	3.85	1.23	0.03	0.24	.89			
I like the idea that I am in control of my experience on the internet	46	4.28	0.78	0.60	0.39	.13			
**Connection seeking**									
Able to help others by sharing my experiences on the Internet	47	3.53	1.08	0.31	0.27	.25			
Help others by sharing my experiences on the Internet	48	3.63	1.14	0.16	0.26	.54			
**Competence Seeking**									
Learn about depression treatment on the Internet	48	3.94	0.78	-0.32	0.38	.41			
Able to learn about the experiences of others on the Internet	48	4.06	0.91	-0.18	0.32	.58			
Helpful to learn about the experiences of others on the Internet	47	3.62	1.17	0.11	0.26	.67			
**Concern for adverse experiences**									
Upset if I read troubling comments about depression on the internet	47	3.21	1.25	-0.16	0.24	.51			
I might read troubling comments about depression on the internet	48	3.67	1.12	-0.58	0.25	**.03**	-0.62	0.28	**.03**
**Preventive health model questions**									
**Beliefs about intervention**									
Participating in a depression Internet social support site is an important thing to do	46	3.59	1.02	0.58	0.29	**.049**	0.27	0.32	.40
A depression Internet social support site makes sense to me	46	3.83	1.06	0.16	0.29	.57			
Depression can be treated successfully	46	4.15	0.87	-0.07	0.35	.85			
Going through a depression internet social support site will help me deal with stress or depression	46	3.50	0.98	0.38	0.31	.22			
**Attitudes toward intervention**									
The benefits of a depression internet social support site outweigh any difficulty	46	3.48	0.86	0.66	0.34	.06			
A depression internet social support site could help me deal with depression	46	3.67	1.06	0.25	0.29	.39			
Help myself overcome depression by visiting a social support Internet site	46	3.37	1.08	0.39	0.28	.17			
Going through a depression Internet social support site is too much trouble	46	2.26	0.80	-0.37	0.38	.34			
A depression internet social support site would be emotionally painful	46	2.17	0.88	-0.23	0.35	.51			
Going to a depression internet social support site would be embarrassing	45	2.04	0.95	0.05	0.33	.87			
I would be better off if I didn't go to this depression Internet support site	46	2.04	0.84	-0.36	0.36	.32			
**Social norms**									
My close friends support me going to depression social support Internet site	45	2.78	1.13	0.17	0.27	.55			
I want to do what my close friends think I should do	45	2.82	0.98	0.24	0.31	.45			
My immediate family supports me going to a depression Internet social support site	45	2.71	1.25	0.30	0.24	.23			
I want to do what the members of my immediate family think I should do	43	2.98	1.03	0.54	0.30	.08			
Physicians and other health care professionals want me to go to an Internet intervention site	45	3.02	1.20	0.47	0.25	.07			
I want to do what the physicians and other health care professionals want me to do	45	3.40	0.91	0.80	0.32	**.02**	0.45	0.36	.23
**Self-efficacy**									
Visiting this depression Internet social support site is an easy thing to do	46	3.89	0.97	0.62	0.30	**.047**	0.43	0.29	.16
Arranging my schedule to visit depression internet social support site would be an easy thing for me to do	46	4.00	0.79	0.35	0.38	.37			
**Transtheoretical Model of Change/MI**									
Importance	48	7.38	2.83	0.14	0.10	.17			

Ability	48	6.81	2.73	-0.11	0.11	.32			
Readiness	48	7.81	2.73	0.04	0.11	.72			
**Participation in decision making**									
Talk to my providers to include my treatment preferences	17	4.12	0.99	0.24	0.47	.61			

Table 3. Predictors of Posts Viewed

				**Model 1**			**Model 2**		
**Predictors**	*N*	*Mean/ (Percent)*	*SD*	*Beta*	*SE*	*P-value*	*Beta*	*SE*	*P-value*
**Mood**									
Baseline PHQ-9	48	13.07	4.44	0.06	0.08	.48			
Baseline CESD	43	9.19	4.64	0.19	0.14	.17			
**Demographics**									
Age	48	36.44	15.98	0.01	0.02	.67			
Male	48	-31.25	-15	0.97	0.75	.20			
College Graduates	48	-41.67	-40	0.78	0.71	.27			
Income	33	45407.7	59496.98	-8.76e	6.00E+00	6.00			
**Social factors**									
Loneliness	34	1.18	0.97	0.2	0.34	.55			
Self-efficacy scale	45	0.44	0.87	0.63	0.37	.09			
**Attitudes toward Internet including Self-Determination Theory**									
**Autonomy seeking**									
My identity to be anonymous while I am exploring concerns on the Internet	47	3.85	1.23	0.62	0.48	.20			
I like the idea that I am in control of my experience on the internet	46	4.28	0.78	-0.25	0.8	.76			
**Connection seeking**									
Able to help others by sharing my experiences on the Internet	47	3.53	1.08	0.04	0.42	.93			
Help others by sharing my experiences on the Internet	48	3.63	1.14	-0.01	0.29	.96			
**Competence Seeking**									
Learn about depression treatment on the Internet	48	3.94	0.78	-0.43	0.41	.29			
Able to learn about the experiences of others on the Internet	48	4.06	0.91	-0.15	0.45	.73			
Helpful to learn about the experiences of others on the Internet	47	3.62	1.17	0.01	0.29	.98			
**Concern for adverse experiences**									
Upset if I read troubling comments about depression on the internet	47	3.21	1.25	9.67E-04	0.39	1.00			
I might read troubling comments about depression on the internet	48	3.67	1.12	-0.18	0.43	.67			

**Preventive Health Model**									
**Beliefs about intervention**									
Participating in a depression Internet social support site is an important thing to do	46	3.59	1.02	0.58	0.49	.24			
A depression internet social support site makes sense to me	46	3.83	1.06	-0.03	0.4	.94			
Depression can be treated successfully	46	4.15	0.87	-0.24	0.64	.71			
Going through a depression internet social support site will help me deal with stress or depression	46	3.5	0.98	0.12	0.47	.81			
**Attitudes toward intervention**									
The benefits of a depression internet social support site outweigh any difficulty	46	3.48	0.86	0.04	0.39	.92			
A depression internet social support site could help me deal with depression	46	3.67	1.06	-0.39	0.5	.44			
Help myself overcome depression by visiting a social support Internet site	46	3.37	1.08	0.09	0.46	.84			
Going through a depression Internet social support site is too much trouble	46	2.26	0.8	0.37	0.43	.40			
A depression internet social support site would be emotionally painful	46	2.17	0.88	-0.17	0.38	.65			
Going to a depression internet social support site would be embarrassing	45	2.04	0.95	0.56	0.37	.13			
I would be better off if I didn't go to this depression Internet support site	46	2.04	0.84	0.31	0.66	.64			
**Social norms**									
My close friends support me going to depression social support Internet site	45	2.78	1.13	0.85	0.4	**.03**	0.13	0.55	.81
I want to do what my close friends think I should do	45	2.82	0.98	0.93	0.53	.08			
My immediate family supports me going to a depression Internet social support site	45	2.71	1.25	0.51	0.34	.13			
I want to do what the members of my immediate family think I should do	43	2.98	1.03	0.3	0.46	.52			
Physicians and other health care professionals want me to go to an Internet intervention site	45	3.02	1.2	0.88	0.33	**.01**	0.79	0.49	.11
I want to do what the physicians and other health care professionals want me to do	45	3.4	0.91	0.65	0.49	.19			
**Self-efficacy**									
Visiting this depression Internet social support site is an easy thing to do	46	3.89	0.97	0.71	0.57	.21			

Arranging my schedule to visit depression internet social support site would be an easy thing for me to do	46	4	0.79	0.15	0.66	.82			
**Transtheoretical Model of Change/MI**									
Importance	48	7.38	2.83	0.16	0.13	.22			
Ability	48	6.81	2.73	0.06	0.15	.68			
Readiness	48	7.81	2.73	0.1	0.13	.43			
**Participation in decision making**									
Talk to my providers to include my treatment preferences	17	4.12	0.99	0.54	0.7	.44			

Table 4. Predictors of Posts Attempted

				**Model 1**			**Model 2**		
**Predictors**	*N*	*Mean/ (Percent)*	*SD*	*Beta*	*SE*	*P-value*	*Beta*	*SE*	*P-value*
**Mood**									
Baseline PHQ-9	48	13.07	4.44	0.32	0.26	.22			
Baseline CESD 10	43	9.19	4.64	0.28	0.15	.06			
**Demographics**									
Age	48	36.44	15.98	0.04	0.04	.33			
Male	48	-31.25	-15	1.46	1.29	.26			
College Graduates	48	-41.67	-40	-0.58	1.23	.64			
Income	33	45407.7	59496.98	-2.28E-05	3.58E-05	.52			
**Social factors**									
Loneliness	34	1.18	0.97	-0.77	0.68	.26			
Self-efficacy scale	45	0.44	0.87	1.56	0.76	**.04**	1.5	0.77	**.05**
**Attitudes toward Internet including Self-Determination Theory**									
**Autonomy seeking**									
My identity to be anonymous while I am exploring concerns on the Internet	47	3.85	1.23	0.13	0.74	.86			
I like the idea that I am in control of my experience on the internet	46	4.28	0.78	-0.41	1.33	.76			
**Connection seeking**									
Able to help others by sharing my experiences on the Internet	47	3.53	1.08	2.05	1.07	.06			
Help others by sharing my experiences on the Internet	48	3.63	1.14	0.45	0.55	.42			
**Competence Seeking**									
Learn about depression treatment on the Internet	48	3.94	0.78	-0.23	0.68	.74			
Able to learn about the experiences of others on the Internet	48	4.06	0.91	0.75	1.05	.47			

Helpful to learn about the experiences of others on the Internet	47	3.62	1.17	0.65	0.58	.26			
**Concern for adverse experiences**									
Upset if I read troubling comments about depression on the internet	47	3.21	1.25	-0.56	0.53	.29			
I might read troubling comments about depression on the internet	48	3.67	1.12	-0.41	0.69	.56			
**Preventive Health Model**									
**Beliefs about intervention**									
Participating in a depression Internet social support site is an important thing to do	46	3.59	1.02	0.22	0.59	.71			
A depression internet social support site makes sense to me	46	3.83	1.06	0.17	0.53	.75			
Depression can be treated successfully	46	4.15	0.87	-1.48	1.29	.25			
Going through a depression internet social support site will help me deal with stress or depression	46	3.5	0.98	1.04	1.05	.32			
**Attitudes toward intervention**									
The benefits of a depression internet social support site outweigh any difficulty	46	3.48	0.86	0.33	0.71	.64			
A depression internet social support site could help me deal with depression	46	3.67	1.06	-0.12	0.74	.87			
Help myself overcome depression by visiting a social support Internet site	46	3.37	1.08	0.17	0.73	.81			
Going through a depression Internet social support site is too much trouble	46	2.26	0.8	0.6	0.89	.50			
A depression internet social support site would be emotionally painful	46	2.17	0.88	-0.45	0.62	.47			
Going to a depression internet social support site would be embarrassing	45	2.04	0.95	0.44	0.53	.41			
I would be better off if I didn't go to this depression Internet support site	46	2.04	0.84	-0.57	0.84	.50			
**Social norms**									
My close friends support me going to depression social support Internet site	45	2.78	1.13	2.04	1.07	.06			
I want to do what my close friends think I should do	45	2.82	0.98	-0.25	1.09	.82			
My immediate family supports me going to a depression Internet social support site	45	2.71	1.25	1.58	0.75	**.04**	1.07	0.57	.06
I want to do what the members of my immediate family think I should do	43	2.98	1.03	0.25	1.08	.81			
Physicians and other health care professionals want me to go to an Internet intervention site	45	3.02	1.2	0.81	0.41	**.046**	0.23	0.43	.60
I want to do what the physicians and other health care professionals want me to do	45	3.4	0.91	1.28	0.67	.06			
**Self-efficacy**									
Visiting this depression Internet social support site is an easy thing to do	46	3.89	0.97	-0.02	1.02	.99			
Arranging my schedule to visit depression internet social support site would be an easy thing for me to do	46	4	0.79	-9.34E-15	1.59	1.00			
**Transtheoretical Model of Change/MI**									

Importance	48	7.38	2.83	0.48	0.36	.18			
Ability	48	6.81	2.73	0.11	0.2	.57			
Readiness	48	7.81	2.73	0.65	0.36	.07			
**Participation in decision making**									
Talk to my providers to include my treatment preferences	17	4.12	0.99	0.94	1.81	.60			
