# Supplementary material for: Internal Versus External Motivation in Referral of Primary Care Patients with Depression to an Internet Support Group: Randomized Controlled Trial
Source: J Med Internet Res. 2013 Mar 12;15(3):e42. doi: 10.2196/jmir.2197 (PMC3636270; doi:10.2196/jmir.2197)
Supplement: Supplementary file 2 [file jmir_v15i3e42_app2.pdf]

CONSORT-EHEALTH Checklist V1.6.2 Report

(based on CONSORT-EHEALTH V1.6), available at [http://tinyurl.com/consort-ehealth-v1-6].

2197

Date completed

5/10/2012 15:15:56

by

Benjamin W. Van Voorhees

Making the Leap from Office to Internet: Randomized Controlled Trial of Internal vs. External Motivation in Referral of Primary Care Patients with Depression to an Internet Support Group and Exploration of Predictors of Use

TITLE

1a-i) Identify the mode of delivery in the title

“Internet Support Group”

1a-ii) Non-web-based components or important co-interventions in title

“Internal vs. External Motivation in Referral”

1a-iii) Primary condition or target group in the title

“Primary Care Patients with Depression”

ABSTRACT

1b-i) Key features/functionalities/components of the intervention and comparator in the METHODS section of the ABSTRACT

“primary care-Internet depression treatment support portal focused around an existent Internet support group (Psycho-Babble)”
“Use of the Internet portal was assessed via automated activity tracking.”

1b-ii) Level of human involvement in the METHODS section of the ABSTRACT

“physician letter of recommendation”

1b-iii) Open vs. closed, web-based (self-assessment) vs. face-to-face assessments in the METHODS section of the ABSTRACT

“identified potential participants using three methods: 1) reading a poster in the waiting room and self-referred; 2) completion of a statement of interest form in their physician’s office after discussion with their physician; and 3) completing a statement of interest at a public information table in a physician office waiting room or in a public location near the clinic. Eligibility was based on a brief structured psychiatric interview based on the Patient Health Questionnaire-9.20 After consent and enrollment, participants were randomly assigned to one of three groups.”

1b-iv) RESULTS section in abstract must contain use data

“Majority of participants were non-White (23/49, 46.94%, African American=20/49, 40.82%, Hispanic=1/49, 2.04%, Asian= 3/49, 6.12%, Other = 2/49, 4.08%) with a mean age of 37.49 (SD=17.15). Forty-one percent (20/49) were college graduates and the mean reported income was $ 45,407 (SD= 59,496). Members of the internal group had more individual data submissions, were more likely to submit at least one element of data, were more likely to have visited with the portal or ISG directly. The internal group was significantly more likely to attempt to submit at least one element of data and had significantly more posts attempted overall and by those who registered. The neutral group spent significantly more time logged into ISG as compared to the external group.”

1b-v) CONCLUSIONS/DISCUSSION in abstract for negative trials

No negative trials

INTRODUCTION

2a-i) Problem and the type of system/solution

“Depressive disorders have a lifetime prevalence of 15% for men and 35% for women. 1,2 Barely half of Americans with a depressive disorder receive any formal treatment and many of those who do receive treatment either do not receive high quality treatment or do not complete a full course of treatment. 3 Chronic care and collaborative care models have demonstrated benefit in improving process of care, symptoms, and functional outcomes 4-6 A key component of these models appears to be support provided by case-management which may act by improving adherence and/or increasing patient activation5,7 However, these models have often proven expensive and cumbersome to implement.6 The rapid development of Internet models that offer self-directed psychotherapy,8 education9 and/or social support10 has offered the prospect for improving depression outcomes in primary care without costly person-to-person interventions or complex organizational change strategies in primary care.”

2a-ii) Scientific background, rationale: What is known about the (type of) system

“Internet sites offering peer-to-peer interaction may be particularly attractive to laypersons as evidenced by wider use of engaging in peer-to-peer and gaming models such as ReachOut in Australia which provides psycho-education with regard to common adolescent mental health problems .12 In the United States, similarly, use of social media to address health concerns is common whereby 28% are estimated to have contacted a support group for a medical condition or personal problem.13 Also, Internet support groups (ISG) have several advantages including: 1) transcending geographic barriers, 2) a lower “risk” disclosure without face-to-face contact, 3) availability of diverse sources of information and 4) a heterogeneous social mix not informed by visual clues.   There is some evidence that use of ISGs for depression may be associated with reductions in depressed mood or the performance of psycho-education functions.”

METHODS

3a) CONSORT: Description of trial design (such as parallel, factorial) including allocation ratio

We conducted a randomized control trial to compare differing three methods of referring primary care patients with depressed mood to an ISG emphasizing “internal” (patient oriented brochure)”, “external” (physician recommendation) and “neutral” (card with Internet address and neither motivational strategies). Also an important consideration was practice burden – interventions that require minimal PCP involvement, such as a paper-based “referral card” with minimal information, but which includes the Internet site address might be particularly useful in actual practice settings. Furthermore, we examined whether baseline attitudes based on the theories of motivational interviewing, Planned Behavior, and as well as self-determination predict Internet site participation.1,19 We hypothesized that 1) primary care referral (all methods) will be effective in engaging participants with the Internet-support group portal (i.e., > 30% of primary care patients would visit the portal); 2) the physician letter recommendation (external) group would have the greatest participation; 3) attitudes toward Internet support group participation would be important predictors of subsequent use; and 4) email reminders to eligible patients would be temporarily associated with first use.”

3b) CONSORT: Important changes to methods after trial commencement (such as eligibility criteria), with reasons

"Several barriers to implementation were addressed after the study was fielded. During the course of the study, two practice sites closed and several physicians subsequently left the practices entirely. After initial attempts using nurses to offer information to patients did not prove successful, physicians were asked to approach appropriate patients directly to determine if they might be interested in the study. The original intention was to have the recommendation letter be from their personal physician; however, the letter was signed by the principal investigator (a primary care physician) instead due to time feasibility limitations. "

3b-i) Bug fixes, Downtimes, Content Changes

"Several barriers to implementation were addressed after the study was fielded. During the course of the study, two practice sites closed and several physicians subsequently left the practices entirely. After initial attempts using nurses to offer information to patients did not prove successful, physicians were asked to approach appropriate patients directly to determine if they might be interested in the study. The original intention was to have the recommendation letter be from their personal physician; however, the letter was signed by the principal investigator (a primary care physician) instead due to time feasibility limitations."

4a) CONSORT: Eligibility criteria for participants

"An eligible participant met the following criteria: 1) PHQ score of 8 or above with either depressed mood or anhedonia and/or is considering treatment for depressed mood, 2) does not reject all treatment for depression, 3) has not viewed or posted messages more than once in the last month on any Internet depression support group website, 4) does not self-report being diagnosed with a bipolar disorder by a health professional, 5) age 18 years or older, 6) attends a primary care clinic and has visited in past 6 months, and 7) has Internet access for the next four weeks, has been on the Internet at least three times, and has used email by him/herself. We excluded those considered to be at high risk for suicide attempts. This included those with past psychiatric hospitalization, past suicide attempts, bipolar disorder or a score of greater than 1 on the PHQ-9 suicide assessment item listed below or who reported intent for self-harm as per assessment (reporting thoughts that you would be better off dead or hurting yourself in some way more than half the days in last two weeks)."

4a-i) Computer / Internet literacy

"has Internet access for the next four weeks, has been on the Internet at least three times, and has used email by him/herself."

4a-ii) Open vs. closed, web-based vs. face-to-face assessments:

"We identified potential participants using three methods: 1) reading a poster in the waiting room and self-referred; 2) completion of a statement of interest form in their physician's office after discussion with their physician; and 3) completing a statement of interest at a public information table in a physician office waiting room or in a public location near the clinic."

4a-iii) Information giving during recruitment

"After consenting to be called for eligibility assessment, each potential participant was called by the study coordinator who conducted a brief structured psychiatric interview based on the Patient Health Questionnaire-9 (PHQ-9).20 After eligibility was confirmed by both the principal investigator (internist-pediatrician) and co-investigator (psychiatrist), participants were offered enrollment in the study."

4b) CONSORT: Settings and locations where the data were collected

"We evaluated several participation outcomes using data obtained from both the portal and the ISG itself (Table 1). We assessed outcomes across several broad categories including: sessions on time, posts viewed and submitted and visitation. We also chose to explore whether the behavior on line varied by virtue of registration on the ISG."

4b-i) Report if outcomes were (self-)assessed through online questionnaires

"We evaluated several participation outcomes using data obtained from both the portal and the ISG itself."

"Socio-demographic factors: We obtained self-reported age, gender, race/ethnicity, and education level. Furthermore, family history of depression or any depressive disorder pertaining to the individual was assessed by the item: "Have you ever been treated for depression (via medication or counseling)?" and "Have any of your family members (mother, father, sister (s), brother (s)) ever been treated for depression that lasted at least four weeks?"

Mood: For the participant's dimensional measures of depressed mood we report Center for Epidemiologic Studies Depression Scale 10 items (CES-D 10, The Cronbach's alpha scale =0.87) 21 and the Patient Health Questionnaire 920 score (The Cronbach's alpha =0.77) .

Self-efficacy: Perceived control was assessed with the Mastery Scale 22 The seven items on the scale measure the extent to which participants see themselves as being in control of the forces that significantly affect their lives with higher scores indicating greater self-efficacy. The Cronbach's alpha for this scale =0.81.

Loneliness: The loneliness item of the CESD-10 was used to report loneliness. Respondents are asked the frequency of feeling lonely in the past week on a 4-point scale (0 = less than a day, 1 = 1-2 days, 2 = 3-4 days, 3 = 5-7 days).

Self-determination theory: Similarly, Self-determination Theory posits that humans seek experiences where they will develop autonomy, connection, and competence. Furthermore, the stronger the "internal motivation" (as opposed to external factors), the higher the quality and enduring the motivation. 1 Based on the focus group, we developed items to address attitudes in each of these domains and one additional one, concern for adverse experiences (See Table 4, Appendix). We measured 9 items based on a Likert-type scale ranging from 1=strongly disagree to 5=strongly agree. The Cronbach's alpha for this scale = 0.69.

Theory of Planned Behavior: We revised the items from a previous questionnaire, which was modified based on the preventive health model and Theory of Planned Behavior 23,24 to the purpose of use of a social support Internet site. According to the Theory of Planned Behavior, intention is the most proximal cognitive measure to actual behaviors. Intention is directly influenced by attitudes and beliefs toward a behavior (e.g., attitudes toward intervention), subjective norms (e.g., concerns with regard to family, peer or employer opinions), perceived behavioral control (i.e., controllability and self-efficacy), influencing performance of a specific behavior (e.g. adhering to the intervention) 19. We measured items based on a Likert-type scale ranging from 1=strongly disagree to 5=strongly agree for items in each domain (See Table 4, Appendix) The reliability (Cronbach's alpha =0.89) and predictive validity of the original instrument has been demonstrated in adolescents.25

Trans-theoretical Model of Change: Items were adapted from Miller and Rollnick's three-item assessment of motivation 26. In terms of validity, this scale and many individual items predicted adherence to an Internet-based depression prevention intervention (modified form).25 Participants rated on 1-10 scale the "importance", their "ability" and their "readiness" to "overcome depressed mood over the next two months."

Shared decision-making: We have previously demonstrated improved outcomes with shared decision making for depression 27. We asked participants to rate agreement with "I can talk with my providers in a way so my preferences for treatment are included" on a 1-5 Likert Scale (strongly disagree to strongly agree)."

4b-ii) Report how institutional affiliations are displayed

"To address these needs, we chose to build a portal (http://www.psycho-babble-study.org) that would: 1) offer access to the fact based information sites (NIMH (http://www.nimh.nih.gov/index.shtml), MOODGym (moodgym.anu.edu.au) and 2) present aspects of the Internet-based support group that focus group participants suggested would be of greatest interest and not initially present those of potential concern or of lower interest."

5) CONSORT: Describe the interventions for each group with sufficient details to allow replication, including how and when they were actually administered

5-i) Mention names, credential, affiliations of the developers, sponsors, and owners

"Dr. Bob's Internet Depression Support Group (Psycho-Babble <http://www.dr-bob.org/babble/>) is a mental health peer support message board that was started by RCH in June 1998."

"Dr. Robert Hsiung operates the Psycho-babble internet sites a limited liability company over which he is the sole proprietor. Revenues from this site are trivial."

5-ii) Describe the history/development process

"Internet Portal: We constructed a portal to connect primary care patients to both fact based information and an established Internet-based support group. Two focus groups identified several key ideas with regard to development of a primary care portal site: 1) offer opportunity to learn from others, 2) offer opportunity to help others, 3) offer fact based resources to evaluate accuracy of information, and 4) protection from disturbing content such as self-harm or salacious topics. To address these needs, we chose to build a portal (http://www.psycho-babble-study.org) that would: 1) offer access to the fact based information sites (NIMH (http://www.nimh.nih.gov/index.shtml), MOODGym (moodgym.anu.edu.au) and 2) present aspects of the Internet-based support group that focus group participants suggested would be of greatest interest and not initially present those of potential concern or of lower interest. The portal allowed participants to view a pre-selected message board and then decide if they wished to progress into actual registration on the Psycho-Babble site. By this method, participants could view online postings while also using the portal to visit other sites.
Psycho-Babble Internet-Based Community: Dr. Bob's Internet Depression Support Group (Psycho-Babble <http://www.dr-bob.org/babble/>) is a mental health peer support message board that was started by RCH in June 1998. Online mental health groups can be classified as autonomous self-help groups or support groups led by mental health professionals. Psycho-Babble is a hybrid that combines the empowerment of self-help with the supportiveness of a milieu maintained by a mental health professional. The asynchronous online (message board) format is highly usable and makes the group more accessible and in some ways safer than groups that meet in person. 35 36 The one original message board has continued to be focused on biological treatments, and additional boards have been added for social support; discussion of the administration of the site, books, psychological treatments, religious faith, complementary and alternative treatments, grief and mourning, substance use, health and wellness, and creative writing."

5-iii) Revisions and updating

"The one original message board has continued to be focused on biological treatments, and additional boards have been added for social support; discussion of the administration of the site, books, psychological treatments, religious faith, complementary and alternative treatments, grief and mourning, substance use, health and wellness, and creative writing."

5-iv) Quality assurance methods

"offer access to the fact based information sites (NIMH (http://www.nimh.nih.gov/index.shtml), MOODGym (moodgym.anu.edu.au)"

5-v) Ensure replicability by publishing the source code, and/or providing screenshots/screen-capture video, and/or providing flowcharts of the algorithms used

5-vi) Digital preservation

""http://www.dr-bob.org/babble/""

5-vii) Access

"The portal allowed participants to view a pre-selected message board and then decide if they wished to progress into actual registration on the Psycho-Babble site. By this method, participants could view online postings while also using the portal to visit other sites."

5-viii) Mode of delivery, features/functionalities/components of the intervention and comparator, and the theoretical framework

"Online mental health groups can be classified as autonomous self-help groups or support groups led by mental health professionals. Psycho-Babble is a hybrid that combines the empowerment of self-help with the supportiveness of a milieu maintained by a mental health professional. The asynchronous online (message board) format is highly usable and makes the group more accessible and in some ways safer than groups that meet in person. 35 36 The one original message board has continued to be focused on biological treatments, and additional boards have been added for social support; discussion of the administration of the site, books, psychological treatments, religious faith, complementary and alternative treatments, grief and mourning, substance use, health and wellness, and creative writing."

5-ix) Describe use parameters

intervention used ad libitum

5-x) Clarify the level of human involvement

"The study was fielded with the initial intent to rely on passive forms of recruitment (information tables and posters) so as to not interfere with the randomization scheme. However, as the study was fielded, it became apparent that direct physician involvement would be required to recruit an adequate sample. Physicians were engaged to offer the study directly to their patients. Consequently, the majority of participants received direct endorsement of the study and portal from their physician before consent and enrollment. Participants were also asked to provide their email so a reminder about the Internet depression site could be emailed to them 2 weeks after enrollment if they had not visited the site."
"A structured approach to managing participant safety was used. After eligibility assessment, all participants with elevated depressed mood received a recommendation for a medical evaluation. All those with self-harm ideation received a structured assessment to determine need for service. While enrolled in the study, the existent Psycho-Babble adverse event reporting mechanisms were developed and were utilized (contact with psychiatrist in case of emergency, safety call assessment by licensed clinical social worker 6-8 weeks after enrollment)."

5-xi) Report any prompts/reminders used

"An email reminder about the Internet depression site was emailed to any participant from any of the above 3 groups who had not visited the site within 2 weeks after enrollment."

5-xii) Describe any co-interventions (incl. training/support)

"As part of this intervention, investigators met with each primary care provider and nurse for 30 minutes during a lunchtime education program. This program included: a) explanation of the study design and the Psycho-Babble Internet site, b) summary of problems in depression care, self-management approaches currently used by individuals with depression, and the state of research on Internet depression support groups. A group of resident physicians received additional training on how to conduct randomized trials as an added inducement to participate in the study."

6a) CONSORT: Completely defined pre-specified primary and secondary outcome measures, including how and when they were assessed

"Outcome Measures
We evaluated several participation outcomes using data obtained from both the portal and the ISG itself (Table 1). We assessed outcomes across several broad categories including: sessions on time, posts viewed and submitted and visitation. We also chose to explore whether the behavior on line varied by virtue of registration on the ISG."

6a-i) Online questionnaires: describe if they were validated for online use and apply CHERRIES items to describe how the questionnaires were designed/deployed

"The main outcome measures were collected in an automated fashion online."
"Independent Variables
Socio-demographic factors: We obtained self-reported age, gender, race/ethnicity, and education level.
Mood: For the participant's dimensional measures of depressed mood we report Center for Epidemiologic Studies Depression Scale 10 items (CES-D 10, The Cronbach's alpha scale =0.87) 21 and the Patient Health Questionnaire 920 score (The Cronbach's alpha =0.77) .
Self-efficacy: Perceived control was assessed with the Mastery Scale 22 The seven items on the scale measure the extent to which participants see themselves as being in control of the forces that significantly affect their lives with higher scores indicating greater self-efficacy. The Cronbach's alpha for this scale =0.81.
Loneliness: The loneliness item of the CESD-10 was used to report loneliness. Respondents are asked the frequency of feeling lonely in the past week on a 4-point scale (0 = less than a day, 1 = 1-2 days, 2 = 3-4 days, 3 = 5-7 days).
Self-determination theory: Similarly, Self-determination Theory posits that humans seek experiences where they will develop autonomy, connection, and competence. Furthermore, the stronger the "internal motivation" (as opposed to external factors), the higher the quality and enduring the motivation. 1 Based on the focus group, we developed items to address attitudes in each of these domains and one additional one, concern for adverse experiences (See Table 4, Appendix). We measured 9 items based on a Likert-type scale ranging from 1=strongly disagree to 5=strongly agree. The Cronbach's alpha for this scale = 0.69.
Theory of Planned Behavior: We revised the items from a previous questionnaire, which was modified based on the preventive health model and Theory of Planned Behavior 23,24 to the purpose of use of a social support Internet site. According to the Theory of Planned Behavior, intention is the most proximal cognitive measure to actual behaviors. Intention is directly influenced by attitudes and beliefs toward a behavior (e.g., attitudes toward intervention), subjective norms (e.g., concerns with regard to family, peer or employer opinions), perceived behavioral control (i.e., controllability and self-efficacy), influencing performance of a specific behavior (e.g. adhering to the intervention) 19. We measured items based on a Likert-type scale ranging from 1=strongly disagree to 5=strongly agree for items in each domain (See Table 4, Appendix) The reliability (Cronbach's alpha =0.89) and predictive validity of the original instrument has been demonstrated in adolescents.25
Trans-theoretical Model of Change: Items were adapted from Miller and Rollnick's three-item assessment of motivation 26. In terms of validity, this scale and many individual items predicted adherence to an Internet-based depression prevention intervention (modified form).25 Participants rated on 1-10 scale the "importance", their "ability" and their "readiness" to "overcome depressed mood over the next two months."
Shared decision-making: We have previously demonstrated improved outcomes with shared decision making for depression 27. We asked participants to rate agreement with "I can talk with my providers in a way so my preferences for treatment are included" on a 1-5 Likert Scale (strongly disagree to strongly agree)."

6a-ii) Describe whether and how "use" (including intensity of use/dosage) was defined/measured/monitored

"We assessed outcomes across several broad categories including: sessions on time, posts viewed and submitted and visitation."

6a-iii) Describe whether, how, and when qualitative feedback from participants was obtained

No qualitative feedback gathered.

6b) CONSORT: Any changes to trial outcomes after the trial commenced, with reasons

No changes.

7a) CONSORT: How sample size was determined

7a-i) Describe whether and how expected attrition was taken into account when calculating the sample size

"A sample size of 225 was estimated to have a power of 0.8 to detect the difference between both the neutral group and the internal or external group (50%, 35% and 10% participation). This sample size was not achieved due to a 9-month delay in study start resulting from an extensive Institutional Review Board review (one year)."

7b) CONSORT: When applicable, explanation of any interim analyses and stopping guidelines

" An interim analysis was conducted for purposes of safety review and grant end of funding. Based on the interim analyses' results which showed significant differences between randomization groups for the main study endpoints and significant predictors for measures of participation, the data monitoring and safety board (DSMB) and the investigators jointly agreed to end the trial."

8a) CONSORT: Method used to generate the random allocation sequence

"(using sealed envelope, equal likelihood of assignment to all arms)"
"We randomly assigned participants (using sealed envelopes) to one of three groups described below without blocking or stratification."

8b) CONSORT: Type of randomisation; details of any restriction (such as blocking and block size)

"(using sealed envelope, equal likelihood of assignment to all arms)"
"We randomly assigned participants (using sealed envelopes) to one of three groups described below without blocking or stratification."

9) CONSORT: Mechanism used to implement the random allocation sequence (such as sequentially numbered containers), describing any steps taken to conceal the sequence until interventions were assigned

"(using sealed envelopes)"
"A data manager not involved in the study execution prepared sealed envelopes."

10) CONSORT: Who generated the random allocation sequence, who enrolled participants, and who assigned participants to interventions

"A data manager not involved in the study execution prepared sealed envelopes."

11a) CONSORT: Blinding - If done, who was blinded after assignment to interventions (for example, participants, care providers, those assessing outcomes) and how

11a-i) Specify who was blinded, and who wasn't

"Participants were aware of group assignments."
"The database manager and the safety caller (described below) were blinded to group assignments."

11a-ii) Discuss e.g., whether participants knew which intervention was the "intervention of interest" and which one was the "comparator"

"We conducted a randomized control trial to compare differing three methods of referring primary care patients with depressed mood to an ISG emphasizing "internal" (patient oriented brochure)", "external" (physician recommendation) and "neutral" (card with Internet address and neither motivational strategies)."
(All were of interest, no group was the comparison.)

11b) CONSORT: If relevant, description of the similarity of interventions

Not relevant.

12a) CONSORT: Statistical methods used to compare groups for primary and secondary outcomes

"Descriptive statistics were calculated for the demographic variables. For between-group categorical comparisons we used the Pearson's chi-square test or the Fisher's exact test when there were < 5 observations per cell. For continuous outcomes, we used analysis of variance (ANOVA) for between group comparisons at the same time points. For continuous between-group data with non-normal distribution, we used the Mann-Whitney test for two-level comparisons. We used a similar analytical approach for comparisons between the three groups and also used the Kruskal Wallis test for three-level comparisons with a non-normal distribution. We developed regression models for three main outcomes: 1) total time on site, 2) number posts viewed, and 3) number posts attempted. Separate regression analyses were performed for the outcome variables. For total time on site the outcome was skewed data and there were values of zero, precluding a logarithmic transformation. The value of 1 was then added to all the variables and they were logarithmically transformed. Linear regression was then performed. For the count data (posts viewed and posts attempted) negative binomial regression was used.   Because of the limited number of observations, we chose to develop only two models for each analysis. Model 1 was a univariate analysis. Model 2 adjusted for all items that were significant or borderline significance for each outcome. STATA 10 was used to conduct the analyses 28.   We also calculated effect sizes for between group comparisons with statistical significance (Cohen's d)."

12a-i) Imputation techniques to deal with attrition / missing values

"With regard to participation outcomes, there was no loss of participants after enrollment."

12b) CONSORT: Methods for additional analyses, such as subgroup analyses and adjusted analyses

None.

RESULTS

13a) CONSORT: For each group, the numbers of participants who were randomly assigned, received intended treatment, and were analysed for the primary outcome

Reported in tables.

13b) CONSORT: For each group, losses and exclusions after randomisation, together with reasons

"With regard to participation outcomes, there was no loss of participants after enrollment."

13b-i) Attrition diagram

No attrition after enrollment.

14a) CONSORT: Dates defining the periods of recruitment and follow-up

"Over the course of the study (November 2008-June 2009)"

14a-i) Indicate if critical "secular events" fell into the study period

"Over the course of the study (November 2008-June 2009)"

14b) CONSORT: Why the trial ended or was stopped (early)

Trial was not stopped early.

15) CONSORT: A table showing baseline demographic and clinical characteristics for each group

Table 2.Demographics
Neutral- Card (A)Internal -Brochure (B) External Recommendation (C)
(Mean)/(SD)(Mean)/(SD)(Mean)/(SD)comparison
Percent NPercent NPercent Np-value
Age{34.83}{12.72}{39.39}{16.83}{34.42}{19.49}0.62
Gender
Male 22.22433.33641.6750.54
Female77.781466.6713*58.337
Ethnicity
White55.561044.44841.6750.71
Non White44.44855.561058.337
Education
Some high school 16.6735.56116.6720.72
High school graduate5.56111.11216.672
Some college27.78538.89741.675
College graduate50.00944.44825.003
Marital Status
Married5.56123.5318.3310.62
Divorced/Separated/Widowed11.11211.7628.331
Never married15.00364.711183.3310
Talk to PCP
Yes61.111158.821027.2730.19
No38.89741.18772.738
Treatment
Yes57.14869.23950.0040.75
No42.86630.77450.004
Family Treatment
Yes27.78547.06816.6720.22
No72.221352.94983.3310
Counseling
Yes55.561070.591250.0060.56
No44.44829.41550.006
Income*{35025.00}{30344.51}{57553.67}{84277.86}{43056.67}{52325.73}0.90
*13 female participants initially enrolled in internal group, but one was unable to complete baseline questionnaire and was not included in subsequent analyses, making final N for group N=18.

15-i) Report demographics associated with digital divide issues

Table 2.Demographics
Neutral- Card (A)Internal -Brochure (B) External Recommendation (C)
(Mean)/(SD)(Mean)/(SD)(Mean)/(SD)comparison
Percent NPercent NPercent Np-value
Age{34.83}{12.72}{39.39}{16.83}{34.42}{19.49}0.62
Gender
Male 22.22433.33641.6750.54
Female77.781466.6713*58.337
Ethnicity
White55.561044.44841.6750.71
Non White44.44855.561058.337
Education
Some high school 16.6735.56116.6720.72
High school graduate5.56111.11216.672
Some college27.78538.89741.675
College graduate50.00944.44825.003
Marital Status
Married5.56123.5318.3310.62
Divorced/Separated/Widowed11.11211.7628.331
Never married15.00364.711183.3310
Talk to PCP
Yes61.111158.821027.2730.19
No38.89741.18772.738
Treatment
Yes57.14869.23950.0040.75
No42.86630.77450.004
Family Treatment
Yes27.78547.06816.6720.22
No72.221352.94983.3310
Counseling
Yes55.561070.591250.0060.56
No44.44829.41550.006
Income*{35025.00}{30344.51}{57553.67}{84277.86}{43056.67}{52325.73}0.90
*13 female participants initially enrolled in internal group, but one was unable to complete baseline questionnaire and was not included in subsequent analyses, making final N for group N=18.

16a) CONSORT: For each group, number of participants (denominator) included in each analysis and whether the analysis was by original assigned groups

16-i) Report multiple “denominators” and provide definitions

"The internal group demonstrated superiority over the external group in several measures of participation (Table 3). Specifically, members of the internal group had more individual data submissions (M=7.53 versus M=0.54, P=0.03, ES=0.60, CI= -0.14, 1.31), were more likely to submit at least one element of data (42.11% versus 7.69%, P=0.05) and were more likely to have visited with the portal or ISG directly (78.95% versus 46.15%, P=0.055). Similarly, the internal group demonstrated superiority over the neutral group with more individual data submissions (M=7.53 versus M=0.39, P=0.01, ES=0.67, CI= -0.02, 1.32). The internal group was significantly more likely to attempt to submit at least one element of data (42.11% versus 5.56%, P=0.02) and had significantly more posts attempted overall and by those who registered (M=5.21 versus M=0.17, P=0.02, ES=0.62, CI= -0.07, 1.27 and M=0.19 versus M=0.01, P=0.02, ES=0.55, CI= -0.19, 1.26 respectively). The neutral group spent significantly more time logged into ISG as compared to the external group (M=9.27 versus M=0.19, P=0.04, ES=0.44, CI= -0.29, 1.15). There was only one three-group comparison that demonstrated significance, which was the percent posting at least once (P=0.02). No other significant differences were detected. "

16-ii) Primary analysis should be intent-to-treat

"The internal group demonstrated superiority over the external group in several measures of participation (Table 3). Specifically, members of the internal group had more individual data submissions (M=7.53 versus M=0.54, P=0.03, ES=0.60, CI= -0.14, 1.31), were more likely to submit at least one element of data (42.11% versus 7.69%, P=0.05) and were more likely to have visited with the portal or ISG directly (78.95% versus 46.15%, P=0.055). Similarly, the internal group demonstrated superiority over the neutral group with more individual data submissions (M=7.53 versus M=0.39, P=0.01, ES=0.67, CI= -0.02, 1.32). The internal group was significantly more likely to attempt to submit at least one element of data (42.11% versus 5.56%, P=0.02) and had significantly more posts attempted overall and by those who registered (M=5.21 versus M=0.17, P=0.02, ES=0.62, CI= -0.07, 1.27 and M=0.19 versus M=0.01, P=0.02, ES=0.55, CI= -0.19, 1.26 respectively). The neutral group spent significantly more time logged into ISG as compared to the external group (M=9.27 versus M=0.19, P=0.04, ES=0.44, CI= -0.29, 1.15). There was only one three-group comparison that demonstrated significance, which was the percent posting at least once (P=0.02). No other significant differences were detected. "

17a) CONSORT: For each primary and secondary outcome, results for each group, and the estimated effect size and its precision (such as 95% confidence interval)

Tables in Appendix.

17a-i) Presentation of process outcomes such as metrics of use and intensity of use

Tables in Appendix.

17b) CONSORT: For binary outcomes, presentation of both absolute and relative effect sizes is recommended

"The internal group demonstrated superiority over the external group in several measures of participation (Table 3). Specifically, members of the internal group had more individual data submissions (M=7.53 versus M=0.54, P=0.03, ES=0.60, CI= -0.14, 1.31), were more likely to submit at least one element of data (42.11% versus 7.69%, P=0.05) and were more likely to have visited with the portal or ISG directly (78.95% versus 46.15%, P=0.055). Similarly, the internal group demonstrated superiority over the neutral group with more individual data submissions (M=7.53 versus M=0.39, P=0.01, ES=0.67, CI= -0.02, 1.32). The internal group was significantly more likely to attempt to submit at least one element of data (42.11% versus 5.56%, P=0.02) and had significantly more posts attempted overall and by those who registered (M=5.21 versus M=0.17, P=0.02, ES=0.62, CI= -0.07, 1.27 and M=0.19 versus M=0.01, P=0.02, ES=0.55, CI= -0.19, 1.26 respectively). The neutral group spent significantly more time logged into ISG as compared to the external group (M=9.27 versus M=0.19, P=0.04, ES=0.44, CI= -0.29, 1.15). There was only one three-group comparison that demonstrated significance, which was the percent posting at least once (P=0.02). No other significant differences were detected. "

18) CONSORT: Results of any other analyses performed, including subgroup analyses and adjusted analyses, distinguishing pre-specified from exploratory

Not done.

18-i) Subgroup analysis of comparing only users

Not done.

19) CONSORT: All important harms or unintended effects in each group
"Fourteen of 49 participants received follow-up calls from the principal investigator related to concerns identified by the assessment caller (depressed mood or self-harm ideation). No study participant reported adverse experiences while on the Internet-support group and there were no self-harm events."

19-i) Include privacy breaches, technical problems
None.

19-ii) Include qualitative feedback from participants or observations from staff/researchers
"No study participant reported adverse experiences while on the Internet-support group and there were no self-harm events."

DISCUSSION

20) CONSORT: Trial limitations, addressing sources of potential bias, imprecision, multiplicity of analyses

20-i) Typical limitations in ehealth trials
"However, several key limitations should be recognized: A) the sample was quite small and the smallest group had only 13 participants, B) a personal letter of recommendation may have performed better than a generic one, C) the closure of two clinics during the study disrupted recruiting and in some cases delivery of the intervention elements, and D) delivery of emails to all groups and similarly direct physician recruitment of nearly all participants may have resulted in reduced between group differences in participation."

21) CONSORT: Generalisability (external validity, applicability) of the trial findings

21-i) Generalizability to other populations
"This study was completed in a variety of primary care settings, which while affiliated with a university, included a broad sample of participants, particularly ethnic minorities. These sites reflected the challenges of contemporary primary care in that two of the practice site closed during the study and were relocated as a result of the financial crisis of 2008. In terms of internal validity, the participants were effectively randomized with no significant between group differences and received three distinct interventions. The sample size while small demonstrated between group differences and helped understand preliminary prediction associations."

21-ii) Discuss if there were elements in the RCT that would be different in a routine application setting
"This study was completed in a variety of primary care settings, which while affiliated with a university, included a broad sample of participants, particularly ethnic minorities. These sites reflected the challenges of contemporary primary care in that two of the practice site closed during the study and were relocated as a result of the financial crisis of 2008. In terms of internal validity, the participants were effectively randomized with no significant between group differences and received three distinct interventions. The sample size while small demonstrated between group differences and helped understand preliminary prediction associations."

22) CONSORT: Interpretation consistent with results, balancing benefits and harms, and considering other relevant evidence

22-i) Restate study questions and summarize the answers suggested by the data, starting with primary outcomes and process outcomes (use)
"Our results support hypothesis 1 that referral of primary care patients to an Internet-based social support group portal was effective (>30% visited site in all groups). Hypothesis 2 was not supported whereby a patient oriented brochure primarily focused on eliciting internal motivations demonstrated greater participation on multiple measures with moderate effect sizes than a generic medical letter of recommendation. Support for hypothesis three was attained with the findings that items from several models, but particularly the Theory of Planned Behavior, and in particular social norms, predicted participation. Furthermore, there appeared to be a temporal association between initial engagement as well as subsequent reminder email and first use supporting hypothesis four."

22-ii) Highlight unanswered new questions, suggest future research
"The next step is a randomized clinical trial in primary care setting to determine if referral to Internet-based social support for those with depressive illness improves quality of care, symptoms and function."

Other information

23) CONSORT: Registration number and name of trial registry
"Trial Registration Information: NCT00886730"

24) CONSORT: Where the full trial protocol can be accessed, if available
clintrials.gov

25) CONSORT: Sources of funding and other support (such as supply of drugs), role of funders
"Acknowledgements: Supported by NIMH R-34 R34 MH 073742-01 and NIMH K-08 MH 072918-01A2 (Dr. Van Voorhees salary support during study)"

X26-i) Comment on ethics committee approval
"The University of Chicago Institutional Review Board approved this study."

x26-ii) Outline informed consent procedures
"After consent and enrollment"

X26-iii) Safety and security procedures
"A structured approach to managing participant safety was used. After eligibility assessment, all participants with elevated depressed mood received a recommendation for a medical evaluation. All those with self-harm ideation received a structured assessment to determine need for service. While enrolled in the study, the existent Psycho-Babble adverse event reporting mechanisms were developed and were utilized (contact with psychiatrist in case of emergency, safety call assessment by licensed clinical social worker 6-8 weeks after enrollment)."

X27-i) State the relation of the study team towards the system being evaluated
"Dr. Van Voorhees has served as a consultant for Prevail Health Solutions, Inc., Mevident Inc., Verimed, Inc., and Social Kinetics, Inc. The University of Chicago has granted a no cost license to Mevident, Inc to adapt the CATCH-IT intervention. Dr. Van Voorhees has agreed to support the company working 5.5 days as consultant at $1,000/day.

Dr. Robert Hsiung operates the Psycho-babble internet sites a limited liability company over which he is the sole proprietor. Revenues from this site are trivial."
